# Small non-coding RNA landscape of extracellular vesicles from a post-traumatic model of equine osteoarthritis

**DOI:** 10.3389/fvets.2022.901269

**Published:** 2022-08-08

**Authors:** James R. Anderson, Stine Jacobsen, Marie Walters, Louise Bundgaard, Andreas Diendorfer, Matthias Hackl, Emily J. Clarke, Victoria James, Mandy J. Peffers

**Affiliations:** ^1^Department of Musculoskeletal and Ageing Science, Institute of Life Course and Medical Sciences, University of Liverpool, Liverpool, United Kingdom; ^2^Department of Veterinary Clinical Sciences, University of Copenhagen, Taastrup, Denmark; ^3^TAmiRNA, TAmiRNA GmbH, Vienna, Austria; ^4^School of Veterinary Medicine and Science, University of Nottingham, Loughborough, United Kingdom

**Keywords:** osteoarthritis, extracellular vesicles, small non-coding RNA, synovial fluid, plasma

## Abstract

Extracellular vesicles comprise an as yet inadequately investigated intercellular communication pathway in the field of early osteoarthritis. We hypothesised that the small non-coding RNA expression pattern in synovial fluid and plasma would change during progression of experimental osteoarthritis. In this study, we conducted small RNA sequencing to provide a comprehensive overview of the temporal expression profiles of small non-coding transcripts carried by extracellular vesicles derived from plasma and synovial fluid for the first time in a posttraumatic model of equine osteoarthritis. Additionally, we characterised synovial fluid and plasma-derived extracellular vesicles with respect to quantity, size, and surface markers. The different temporal expressions of seven microRNAs in plasma and synovial fluid-derived extracellular vesicles, eca-miR-451, eca-miR-25, eca-miR-215, eca-miR-92a, eca-miR-let-7c, eca-miR-486-5p, and eca-miR-23a, and four snoRNAs, U3, snord15, snord46, and snord58, represent potential biomarkers for early osteoarthritis. Bioinformatics analysis of the differentially expressed microRNAs in synovial fluid highlighted that in early osteoarthritis these related to the inhibition of cell cycle, cell cycle progression, DNA damage and cell proliferation as well as increased cell viability and differentiation of stem cells. Plasma and synovial fluid-derived extracellular vesicle small non-coding signatures have been established for the first time in a temporal model of osteoarthritis. These could serve as novel biomarkers for evaluation of osteoarthritis progression or act as potential therapeutic targets.

## Introduction

Osteoarthritis (OA) is a degenerative joint disease characterised by deterioration of articular cartilage and accompanied by changes in the bone and soft tissues of the joint (1), which adversely impact the health of equine athletes. It is a major welfare issue resulting in substantial morbidity and mortality (2). Lameness resulting from OA is a major cause of poor performance and early retirement (3). Despite the huge welfare importance of OA, our understanding of pathophysiological mechanisms involved is limited (4). OA is characterised by increase in cartilage extracellular matrix (ECM) degradation by proteases and reduction in ECM production (5). We recently identified that differential expression (DE) of small nucleolar RNAs (snoRNAs) contributes to this imbalance, a key mechanism in OA (6, 7). We require biomarkers to identify early OA before cartilage ECM is irreversibly degraded, and our group has identified small non-coding RNAs (sncRNAs) distinguishing early equine OA synovial fluid (SF) (8). These include microRNAs (miRNAs) and snoRNAs, which are functional RNA molecules that are transcribed from DNA but do not translate into proteins (9).

It is expected that novel therapies can be directed in a personalised manner (10) following disease stratification. This is imperative as OA therapies are currently only symptomatic; principally pain relief in the horse. Extracellular vesicles (EVs) from purified cell types are suggested as novel therapeutics in a number of human diseases including rheumatoid arthritis (11), cardiac disease (12), and neoplasia (13). EVs have the potential to accelerate tissue regeneration and improve tissue functions. In disease treatment, they have been suggested as treatment strategies and delivery systems. Stem cell-derived EVs can recapitulate their parent cells, with advantages of being less immunogenic and capability of crossing biological barriers (14).

We have identified DE sncRNAs in ageing equine and human and OA human cartilage (15, 16), equine OA SF (8), and mouse serum (17, 18). In human OA, others have identified sncRNAs in joint tissues, plasma, and SF as potential therapeutics (19, 20) or possible OA biomarkers (18, 21–24). EVs produced by cells transfer molecules (including sncRNAs) between cells and tissues (25) and are known to be found in serum, SF, articular cartilage, and supernatants of synoviocytes and chondrocytes (26). EV cargo is involved in cross-talk between cells in joint tissues and affects ECM turnover and inflammation (27, 28), representing a crucial step in OA regulation. The role of EVs in OA provides a foundation to create novel disease-modifying treatments (27). Promising results were obtained in therapeutic application of mesenchymal stem cell-derived EVs for cartilage repair and experimental OA (29). Additionally, EVs have a therapeutic potential in human rheumatoid arthritis when administered either into the joint or systemically (27). Whilst EVs can carry various types of cargo, increasing evidence points at miRNAs as considerable mediators for the effects of these vesicles on their target cells (30, 31). Significantly, miRNAs regulate signalling pathways and the immune system related to extracellular matrix synthesis, chondrocyte survival, and proliferation (32, 33).

Animal models of OA enable the reproduction and progression of degenerative damage to be measured in a controlled manner, enabling opportunities to monitor and modulate symptoms and disease progression (34). Although the equine carpal osteochondral fragment model (35, 36) does not encompass all pathophysiological aspects of different OA phenotypes, it allows a controlled system with known time of onset and a singular cause of OA, facilitating temporal OA progression studies. Molecules can be measured temporally to determine their role in early OA. This is particularly imperative as the precise onset of molecular events of early OA following trauma is not completely understood. Furthermore, the size of the equine middle carpal joint permits repeated SF sampling over time, thus limiting use of experimental animals. Thus, the carpal fragment model enables a simultaneous study on equine OA with the ability to apply the findings to understand human OA.

We hypothesise that the EV sncRNA cargo from SF and plasma can be used to identify OA in an early stage before clinical signs and irreversible cartilage degradation. Additionally, by determining changes in sncRNAs in a longitudinal manner, we may be able to further understand the pathogenesis of early OA.

## Materials and methods

### Horses and study design

The Danish Animal Experiments Inspectorate (permit 2017-15-0201-01314) and local ethical committee of the Large Animal Teaching Hospital of University of Copenhagen approved the experimental protocol. All procedures were undertaken according to EU Directive 2010/63/EU for animal experiments.

Four skeletally mature standard-bred trotters (2.5–7 years old, weighing 397–470 kg) were included in this study. Prior to inclusion, the horses underwent clinical examination, lameness examination including flexion tests, radiographic imaging, haematological and blood-biochemical analysis, and arthrocentesis of both middle carpal joints to ensure that the horses were sound with healthy joints.

OA was surgically induced in the left middle carpal joint, and the right middle carpal joint underwent sham surgery as described previously (36). Plasma and SF were sampled from both middle carpal joints before and following OA induction as described below.

The horses were euthanised on day 71/72 with pentobarbital sodium (140 mg/kg, Euthasol Vet, Dechra Veterinary Products, Uldum, Denmark). Following euthanasia, samples were collected from the joints as detailed below.

### Induction of osteoarthritis and exercise

OA induction was undertaken as described by McIlwraith (36) under general anaesthesia. In brief, an osteochondral fragment was created in the third facet of the radial carpal bone with an 8-mm curved osteotome, and the fragment was left in the joint partially attached to the parent bone. Sham surgery (arthroscopy alone) was performed on the right middle carpal joint. Two weeks following the OA induction, the horses were made to exercise with 2 min of trot (4.4–5.3 m/s), 2 min of fast trot/gallop (9 m/s), and 2 min of trot (4.4–5.3 m/s) for 5 days/week on a treadmill (37).

### SF and plasma sampling

SF samples were obtained from both middle carpal joints prior to (day 0) and 10, 35, 42, 49, 56, and 63 days after the surgery. However, the samples of SF and plasma were taken at additional time points prior to day 10 and on day 70 these had been used in previous studies. Therefore, we used time points where we had sufficient volumes for our experiments. Following sedation with detomidine (0.01 mg/kg, Domosedan Vet; Orion Pharma Animal Health, Copenhagen, Denmark) and butorphanol tartrate (0.01 mg/kg, Dolorex; MSD Animal Health, Copenhagen, Denmark), SF was aspirated aseptically with a 19-gauge, 40-mm needle into ethylene diamine tetra acetic acid (EDTA) tubes (BD Vacutainer, BD A/S, Albertslund, Denmark). These were inverted 5–10 times, kept on melting ice, and centrifuged at 1,000 *g* for 20 min at 4°C. Plasma was collected at the same time points following intravenous sampling from an indwelling catheter in the jugular vein on days 0–14 and thereafter by venipuncture. Samples were collected directly into EDTA tubes, kept on melting ice, and centrifuged at 3,000 *g* for 15 min at 4°C. Biofluids were processed within 1 h and stored at −80°C immediately after centrifugation.

### Post-mortem examination

Following euthanasia, the middle carpal joints were opened, and the synovial membrane and articular cartilage were obtained from the intermediate carpal and third carpal bone and placed in neutral buffered 10% formalin. For histology, the samples were processed for haematoxylin and eosin and safranin O (cartilage only) staining. Histological grading of the synovial membrane and cartilage was performed (38).

### EV isolation

The SF and plasma collected on days 0, 10, 35, 42, 49, 56, and 63 from OA and control joints were thawed, and SF was subsequently treated with 1 μg/ml hyaluronidase (from bovine testes; Sigma-Aldrich, Gillingham, United States). Both the SF and the plasma were centrifuged at 2,500 *g* for 10 min and 10,000 *g* for 10 min at 4°C. EVs were subsequently isolated by size exclusion chromatography using qEV single columns (IZON, Lyon, France) following the manufacturer's instructions. Briefly, 3.5 ml of phosphate buffered saline (PBS; Sigma-Aldrich, Gillingham, United Kingdom), previously processed using a 0.22-μm polyethersulfone philtre (Sartorius, Göttingen, Germany) was passed through the column, followed by 150 μl of SF or plasma. The first five 200 μl that flowed through fractions were discarded, and the following five 200 μl fractions were pooled (isolated EVs).

### EV characterisation

#### Nanoparticle tracking analysis

For all the samples, 100 μl of isolated EVs were characterised by nanoparticle tracking analysis (NTA; Nanosight NS300; Malvern Panalytical Ltd., Malvern, United Kingdom) at 25°C to determine particle size and EV concentration (39). Camera level, slider shutter, and slider gain were set at 12, 1,200, and 146, respectively. For each sample, three replicate 60-s videos were recorded and the results were averaged. For analysis, detection threshold was set at 5. Data analysis was performed with the NTA 3.2 software (Malvern Panalytical, Malvern, United Kingdom). A statistical analysis comparing the groups was conducted using mixed effects models with the Dunnett's and Sidak's multiple comparisons tests applied to plasma and SF-derived EVs, respectively, using GraphPad Prism v8.0.1. Significant differences between the groups were identified at *p* ≤ 0.05.

#### Exoview

SF and plasma EV isolates collected on days 0, 42, and 63 were concentrated using 2 ml Vivaspin concentrator columns (100,00 kDa cutoff) (Sartorius, Göttingen, Germany) by centrifuging at 1,000 *g* until the final volume was 100 μl and subsequently centrifuged into a column cap at 4,000 *g* for 2 min. Five μl of each sample was removed and pooled with all corresponding samples of the same group and time point. This single pooled sample from n = 4 horses was then analysed, with a total of six samples (day 0 control, day 0 OA, day 42 control, day 42 OA, day 63 control, and day 63 OA). ExoView analyses EVs using visible light interference in order to determine EV size, in addition to fluorescence to measure specific immunocaptured proteins. The samples were analysed in technical triplicate/sample using the human ExoViewTetraspanin Kit (NanoView Biosciences, United States). The samples were diluted in a manufacturer-supplied incubation solution and incubated overnight at room temperature on ExoViewTetraspanin Chips. The chips were washed three times in solution A prior to incubation with fluorescent tetraspanin antibodies. Labelling antibodies consisted of anti-CD9 CF488, anti-CD81 CF555, and anti-CD63 CF647 and the MIgG negative control. The antibodies were diluted to 1:500 as per the manufacturer's instructions and incubated on the chips for 1 h at room temperature. The chips were then washed in kit-supplied buffers, dried, and imaged with ExoView R100 using ExoView Scanner v3.0. Data were analysed using ExoView Analyzer v3.0. Fluorescent cut-offs were set relative to the MIgG control. Total EVs were determined as the number of detected particles bound to tetraspanin antibodies (CD9, CD81, and CD63) and normalised to MIgG antibody. Particle diameter and counts were statistically analysed by repeated measures ANOVA in GraphPad Prism v9.0.1 and Excel. Significant differences between the groups were identified at *p* ≤ 0.05.

### Small RNA sequencing

#### RNA isolation, library preparation for small RNA-Seq, and sequencing

Total RNA was extracted from 200 μl of isolated EVs using the miRNeasy Serum/Plasma Advanced Kit (Qiagen, Crawley, United Kingdom) and following the manufacturer's instructions, including supplementation of an RNA spike-in (Qiagen, Crawley, United Kingdom) and on-column DNase treatment (Qiagen, Crawley, United Kingdom). In addition, glycogen (Thermo Scientific, Waltham, Massachusetts, United States) was added to a final concentration of 50 pg/ml prior to adding isopropanol. Because of low RNA yield, which impairs accurate determination of RNA concentrations, a fixed total RNA volume of 2 μl was used for library preparation using a CleanTag kit (TriLink, San Diego, United States). Adapter dilution (1:4) and PCR amplification (26 cycles) were optimised in a pilot experiment using Bioanalyzer DNA1000 (Agilent, Santa Clara, United States) chips as a read out of library size and quantity. The samples were then processed in two batches of 37 samples. Library yield and size range were confirmed for all the samples using Bioanalyzer DNA1000 chips (Agilent, Santa Clara, United States). Equimolar amounts of all the samples were pooled, and size purification was performed using BluePippin (Sage Biosystems, Beverly, United States). To maintain larger RNA fragments, the size range was extended to 130–300 bp, which corresponds to insert sizes between 15 and 180 bp. The purified pool was sequenced on a NovaSeq SP100 flow cell (Illumina, San Diego, United States).

#### Small RNA sequencing data processing

Data were analysed using the miND pipeline (40). The overall quality of the next-generation sequencing data was evaluated automatically and manually with fastQC v0.11.9 (41) and multiQC v1.10 (42). Reads from all passing samples were adapter-trimmed and quality-filtered using cutadapt v3.3 (43) and filtered for a minimum length of 17 nt. Mapping steps were performed with bowtie v1.3.0 (44) and miRDeep2 v2.0.1.2 (45), whereas the reads were mapped first against the genomic reference EquCab.3.0 provided by Ensembl (46), allowing for two mismatches and, subsequently, miRBase v22.1 (47), filtered for miRNAs of *Equus caballus* only, allowing for one mismatch. For a general RNA composition overview, non-miRNA mapped reads were mapped against RNAcentral (48) and then assigned to various RNA species of interest. A statistical analysis of pre-processed next-generation sequencing data was conducted with R v4.0 and the packages pheatmap, pcaMethods v1.82, and genefilter v1.72. A DE analysis with edgeR v3.32 (49) used the quasi-likelihood negative binomial generalised log-linear model functions provided by the package. The independent filtering method of DESeq2 (50) was adapted for use with edgeR to remove low abundant miRNAs and thus optimise false discovery rate (FDR) correction. Significantly, DE sncRNAs were determined at *p* < 0.05.

#### Bioinformatics of differentially expressed miRNAs

Potential biological associations of the DEmiRNAs in EVs derived from plasma or SF were identified using Ingenuity Pathway Analysis (IPA; Qiagen, Redwood City, CA, United States) “Core Analysis.” Furthermore, to identify putative miRNA targets, we utilised the MicroRNA Target Philtre module within IPA. We used a conservative filter within IPA of experimentally validated and highly conserved predicted mRNA targets for each miRNA. ToppGene was used for functional enrichment analysis of the miRNA targets (51) with a Bonferroni FDR <0.05. Biological process gene ontology (GO) terms generated through ToppGene were then summarised, and REViGO (52) and Cytoscape (53) were used to visualise the network.

## Results

### Model outcome

As previously described, the end point synovial membrane scores were significantly increased, with increased cellular infiltration, intimal hyperplasia, subintimal oedema, and increased total final histological score in the OA joints vs. control joints (*p* < 0.05). Additionally, a previous histological evaluation of the third carpal bone cartilage demonstrated a significant increase in chondrocyte necrosis, cluster formation, and focal cell loss scores in OA joints, and a significant increase in final score (*p* < 0.05) (54). Representative examples of gross photographs of a control carpal joint and a carpal joint following OA induction plus representative histological sections are shown in Supplementary material 1.

### EV characterisation

#### Nanoparticle tracking analysis

**Figure 1:**
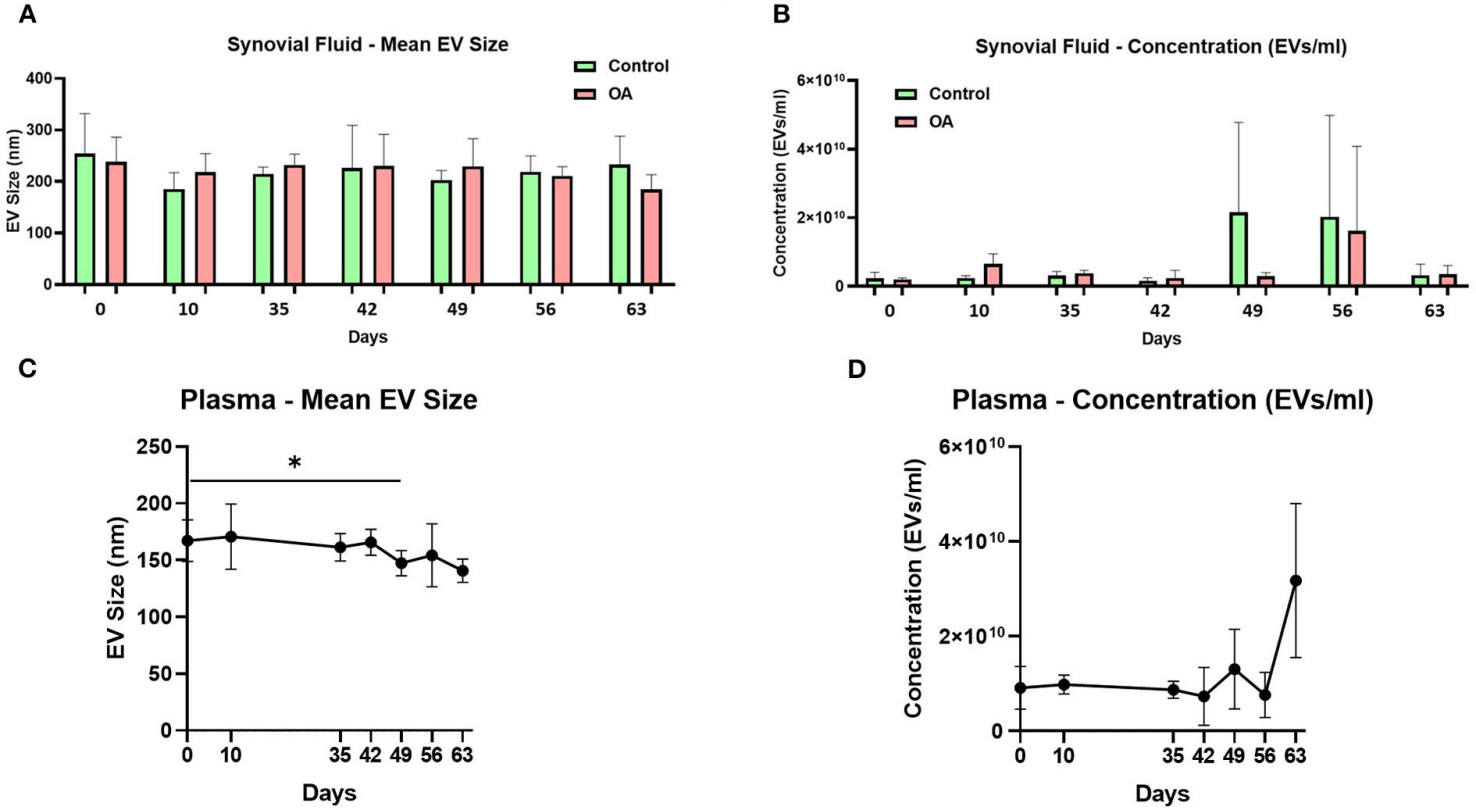
Synovial fluid and plasma-derived extracellular vesicle size and concentration measured by nanoparticle tracking. **(A)** Mean size of extracellular vesicles isolated from synovial fluid control (green) and osteoarthritic (red) middle carpal joints prior to surgery (day 0) and on days 35, 42, 49, 56, and 63 following model induction. **(B)** Mean concentration of isolated extracellular vesicles in the synovial fluid. Synovial fluid samples were measured from all the horses (*n* = 4) at all time points for control and OA joints. **(C)** Mean size of extracellular vesicles isolated from the plasma prior to surgery (day 0) and on days 35, 42, 49, 56, and 63 following model induction. **(D)** Mean concentration of isolated extracellular vesicles in the plasma. Plasma samples from all the horses (*n* = 4) were measured at all time points. All the analyses were conducted by nanoparticle tracking using Nanosight NS300. Error bars ± 1 standard deviation. *p < 0.05.

The mean and mode particle size for control SF were 219.8 and 180.9 nm, OA SF 221.8 and 195 nm, and plasma 158.4 and 123.3 nm. We did not identify differences for SF-derived EVs in size, size distribution, or concentration between the control and OA joints. However, for plasma-derived EVs, a reduction in the mean EV size was identified on day 49 compared to day 0, and a reduction in D90 was identified on day 63 compared to day 0. No changes in plasma EV concentrations were identified over time (Figure 1 and Supplementary material 2).

#### Exoview

**Figure 2:**
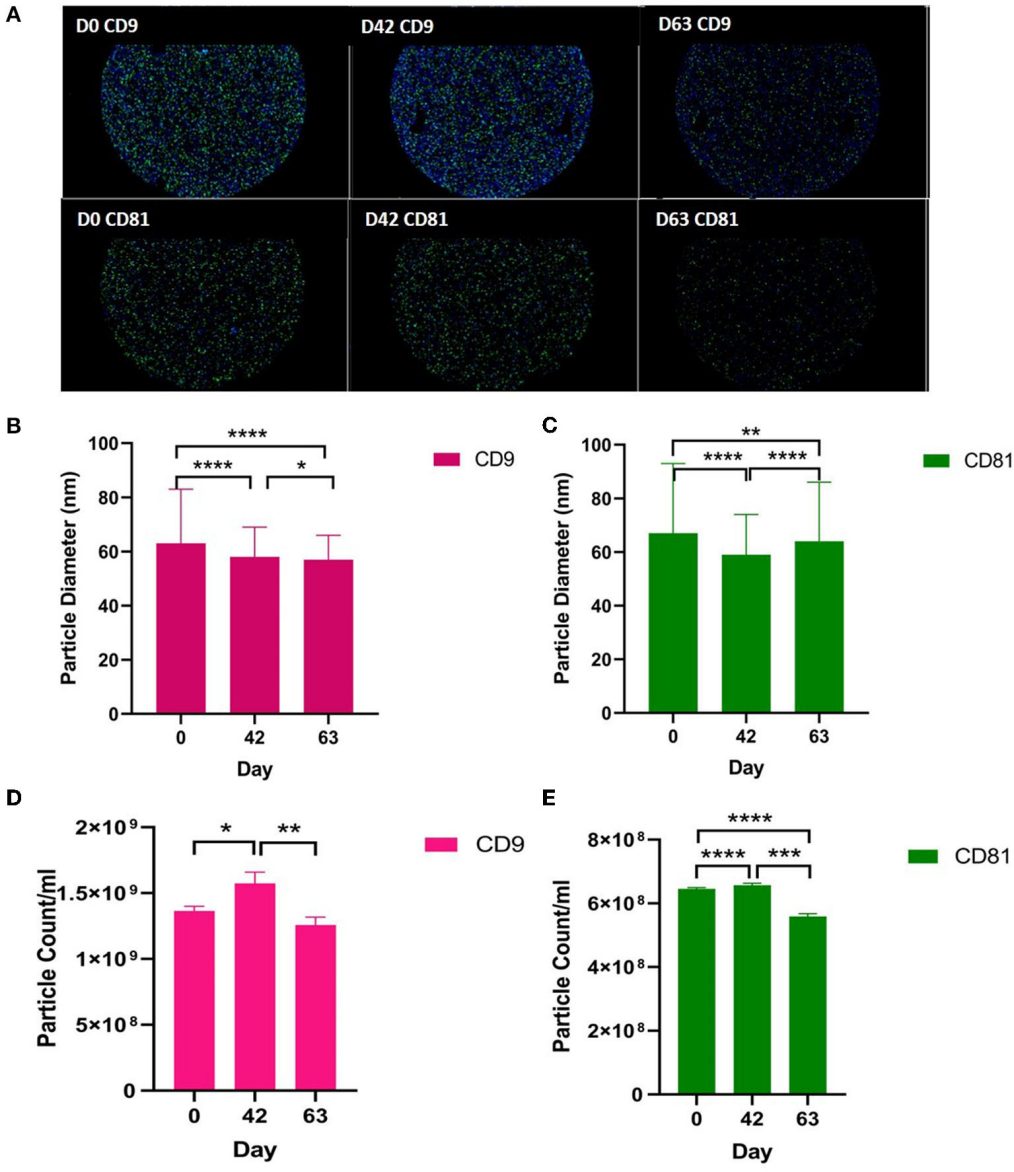
Visualisation, sizing, and enumeration of plasma-derived EVs using Exoview at selected time points. All the data were adjusted for dilution of the sample onto the chip. Average of three technical replicates was run. Particle number was quantified by the number of particles in a defined area on the antibody capture spot. All bars are means and error bars standard deviation. Each sample represents a pool of all the four horses at prior to induction (day 0) and days 42 and 63 after the induction of OA. **(A)** Fluorescent image of a representative spot for each sample. **(B,C)** Sizing of **(B)** CD9- and **(D)** CD81-labelled EVs normalised to MIgG control. Limit of detection was 50–200 nm. **(D,E)** Counting of CD9- and CD81-positive particles after probing with fluorescent tetraspanin antibodies. Statistical analysis was conducted in GraphPad Prism 9.0 by *t*-tests following parametric evaluation (*p < 0.05, **p < 0.01, ***p < 0.001, ****p < 0.0001).

**Figure 3:**
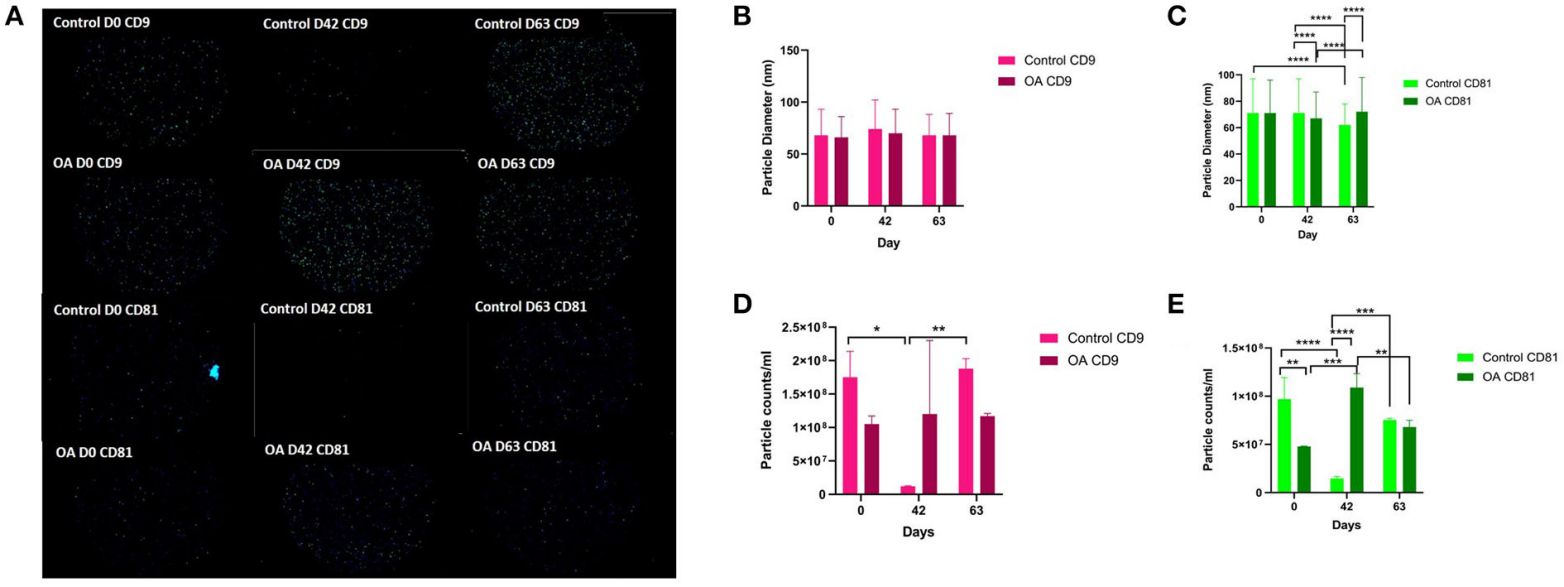
Visualisation, sizing, and enumeration of SF-derived EVs from control and osteoarthritic (OA) joints using Exoview at selected time points. All the data were adjusted for dilution of the sample onto the chip. Average of three technical replicates was run. Particle number was quantified by the number of particles in a defined area on the antibody capture spot. All bars are means and error bars standard deviation. Each sample represents a pool of all the four horses prior to induction (day 0) and on days 42 and 63 after induction of OA **(A)** Fluorescent image of a representative spot for each sample. **(B,C)** Sizing of **(B)** CD9- and **(C)** CD81-labelled EVs normalised to MIgG control. Limit of detection was 50–200 nm. **(D,E)** Counting of **(D)** CD9- and **(E)** CD81-positive particles after probing with fluorescent tetraspanin antibodies. Statistical analysis was conducted in GraphPad Prism 9.0 by repeated measures ANOVA (*p < 0.05, **p < 0.01, ***p < 0.001, ****p < 0.0001).

We analysed the vesicle count, size, and heterogeneity of SF and plasma EVs using Exoview in accordance with MISEV guidelines (55) in order to validate that the particles isolated were EVs. The human ExoView chip assessed CD9, CD63, and CD81 markers. For all the equine samples, there was minimal binding on CD63 capture, suggesting low-sequence homology between human and equine CD63. CD63 data were therefore excluded from further analysis. Particles stained for CD9 and CD81 were more obvious in plasma-derived EVs (Figure 2A) because of the higher concentration of EVs compared to SF (~1.8 times as concentrated in plasma-derived EVs) (Figure 3A). The plasma EVs had mean diameters between 57 and 67 nm (Figures 2B,C), and SF 62–74 nm (Figures 3B,D), both being dependent on the tetraspanin subpopulation. The diameter of the temporal population of plasma-derived EVs changed significantly for both CD9-positive (Figure 2C) and CD81-positive (Figure 2D) EVs following the OA induction. The number of plasma-derived EVs peaked significantly on day 42 for both CD9-postive EVs (Figure 2D) and CD81-positive EVs (Figure 2E) following the OA induction. Interestingly in the SF, there was no difference in vesicle diameter with time or OA for CD9 (Figure 3B). However, there were differences in temporal and disease-related expressions of CD81-captured EVs in the SF (Figure 3C). There were statistical differences in temporal expression of CD9 EVs in the control but not in the OA samples (Figure 3D). For CD9 and CD81 in the control samples only, expression was lowest on day 42 (Figures 3D,E). The expression of CD81-labelled vesicles was significantly different between OA and control on days 0 and 42 in the SF (Figure 3E). From the co-localisation results of CD9 and CD81, there was an apparent drop-off in the proportion of EVs double positive at the final time point on day 63 in the plasma (Supplementary material 3).

### Small RNAseq results

In the SF and plasma-derived EVs, we identified multiple classes of non-coding RNAs including miRNAs, snoRNAs, small nuclear RNAs (snRNAs), transfer RNAs (tRNAs), long non-coding RNAs (lncRNAs), y-RNAs, piwi RNAs (piRNAs), and scaRNAs (scRNA). Supplementary material 4 shows the number of each class of miRNA, snoRNA, snRNA, tRNA, lncRNA that was identified in at least 30% of the samples in that group (plasma or synovial fluid-derived EVs).

#### miRNAs

**Figure 4:**
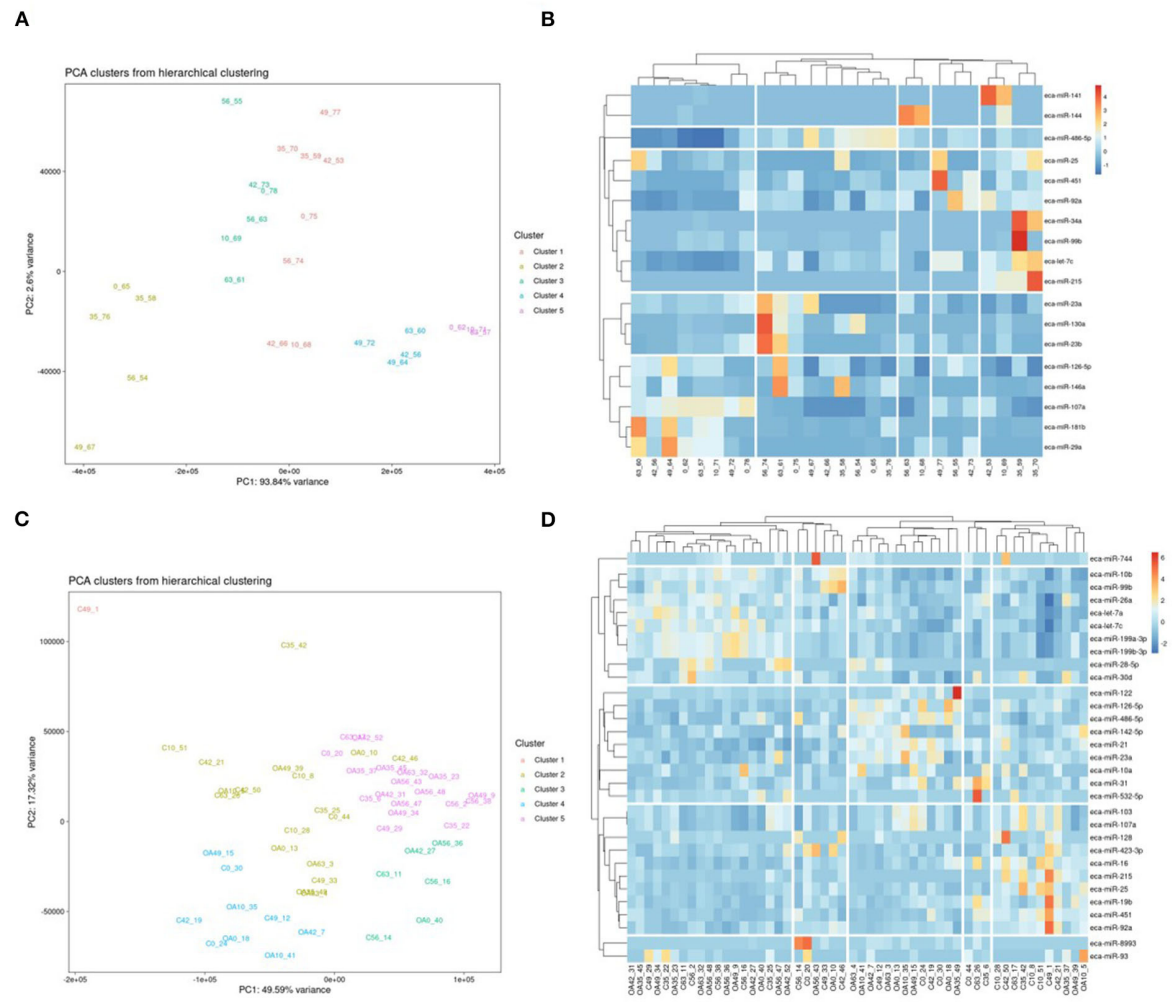
Summary of data following small noncoding RNA sequencing of plasma and synovial fluid-derived EVs. **(A)** PCA of differentially expressed (DE) miRNAs for plasma-derived EVs. **(B)** Heatmap of DE miRNAs (*p* < 0.05) in plasma-derived EVs. **(C)** PCA of miRNAs for synovial fluid-derived EVs (C, control; OA, osteoarthritis). Numbering of samples: the first number is the day of sampling, 0, 10, 35, 42, 49, 56, and 63. The second number is the original sample ID (*n* = 4 horses). **(D)** Heatmap of DE miRNAs (*p* < 0.05) in synovial fluid-derived EVs. Heatmaps were created using the R pheatmap package. Clustering was conducted with the “average” method and by correlation distancing. Scaling is unit variance scaled expression and based on read counts. Clustering distances are based on Pearson correlation.

**Figure 5:**
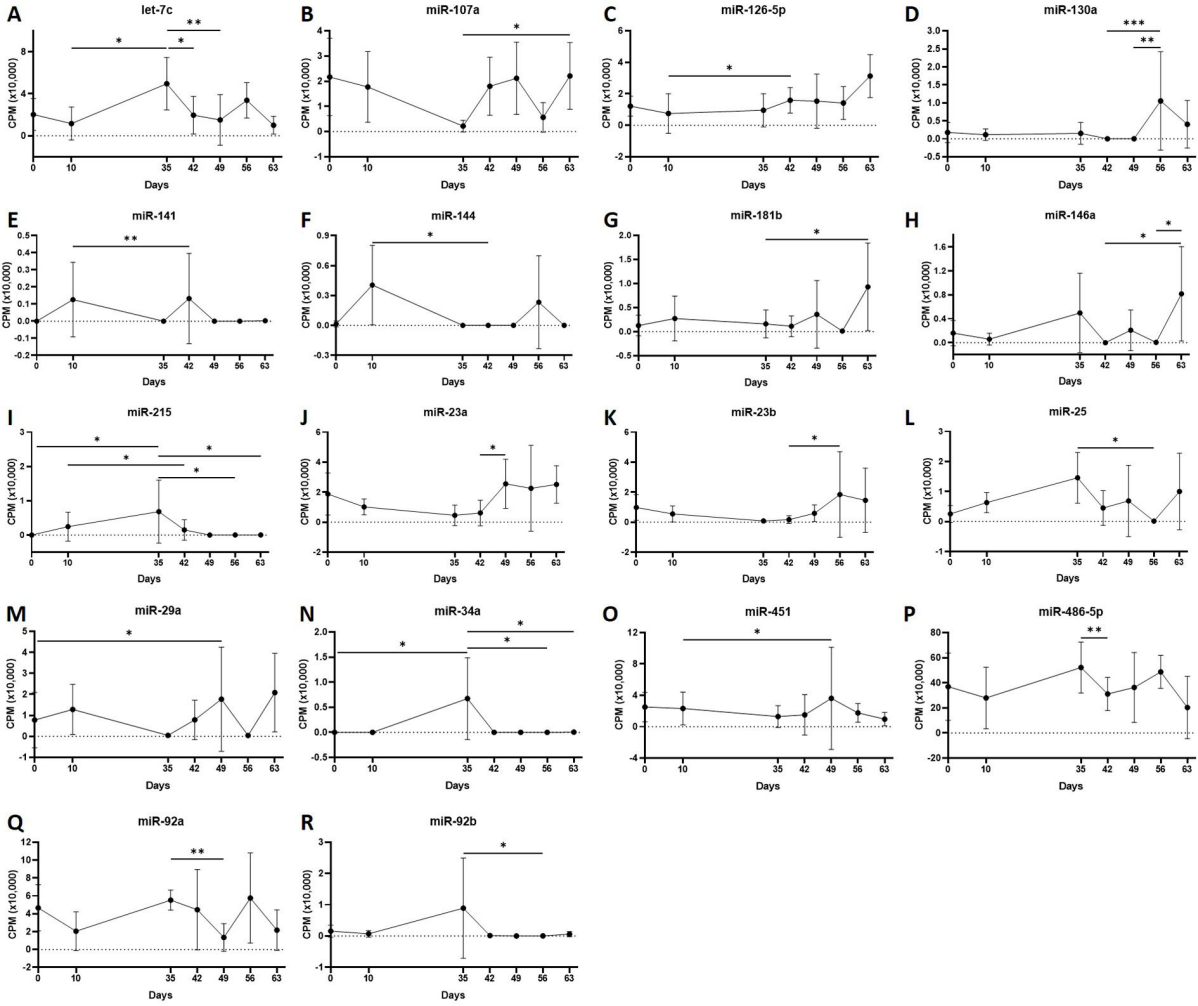
**(A–R)** Differentially expressed miRNAs isolated from plasma-derived extracellular vesicles following small RNA sequencing. Individual panels for 18 miRNAs differentially expressed using pairwise comparisons of all the six time points (days 0, 35, 42, 49, 56, and 63), miR, eca-miR, *n* = 4 horses. Error bars ± 1 standard deviation. *p < 0.05, **p < 0.01, and ***p < 0.001.

**Figure 6:**
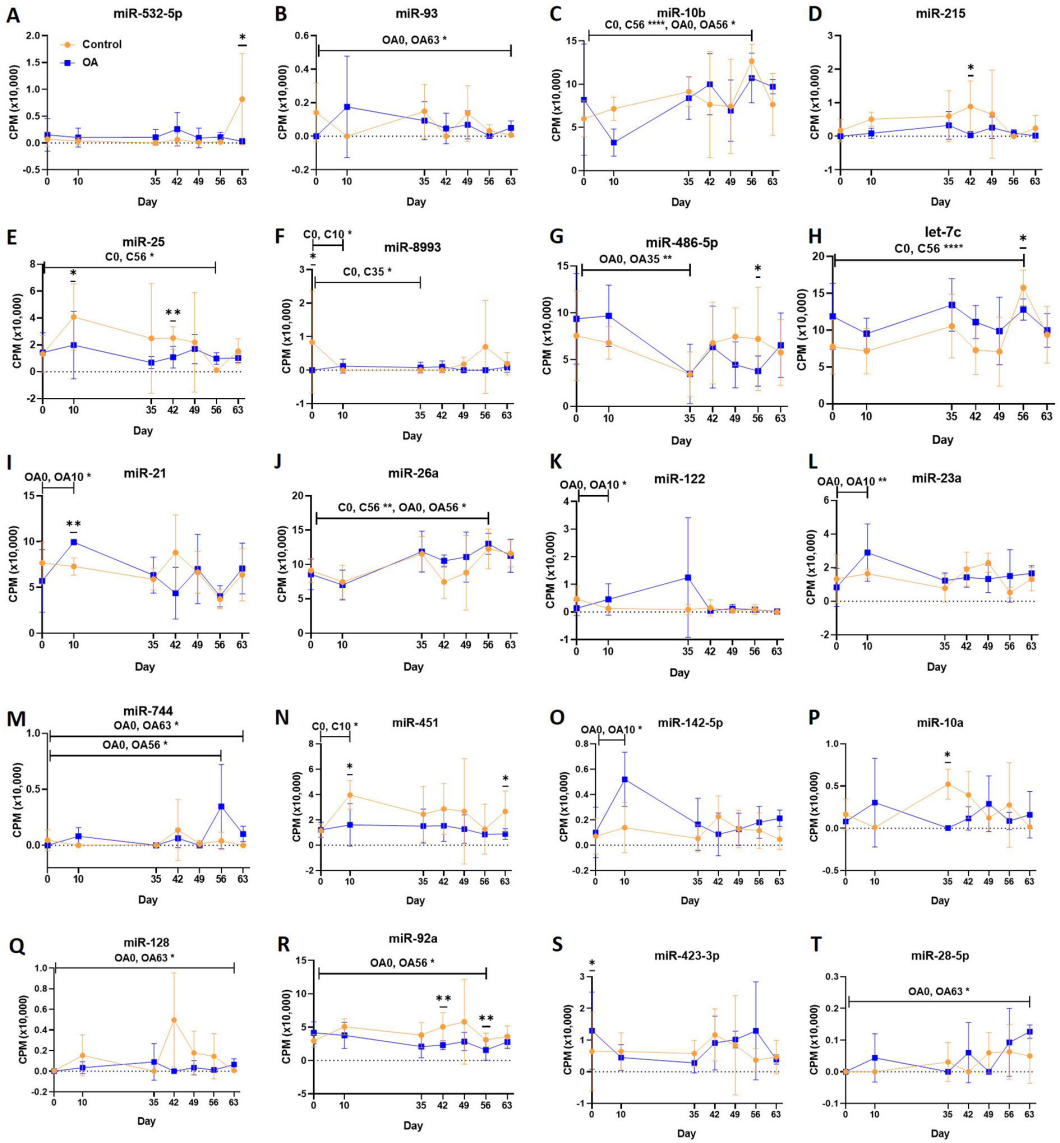
**(A–T)** Differentially expressed miRNAs isolated from synovial fluid-derived extracellular vesicles following small RNA sequencing. Comparisons were made between control (C) and osteoarthritis (OA) and between OA time points (days 0, 10, 35, 42, 49, 56, and 63), miR; eca-miR, *n* = 4 horses. Individual panels for the 20 DE miRNAs altered between OA time points or between control and OA. Error bars ± 1 standard deviation. *p < 0.05, **p < 0.01, and ****p < 0.0001.

**Figure 7:**
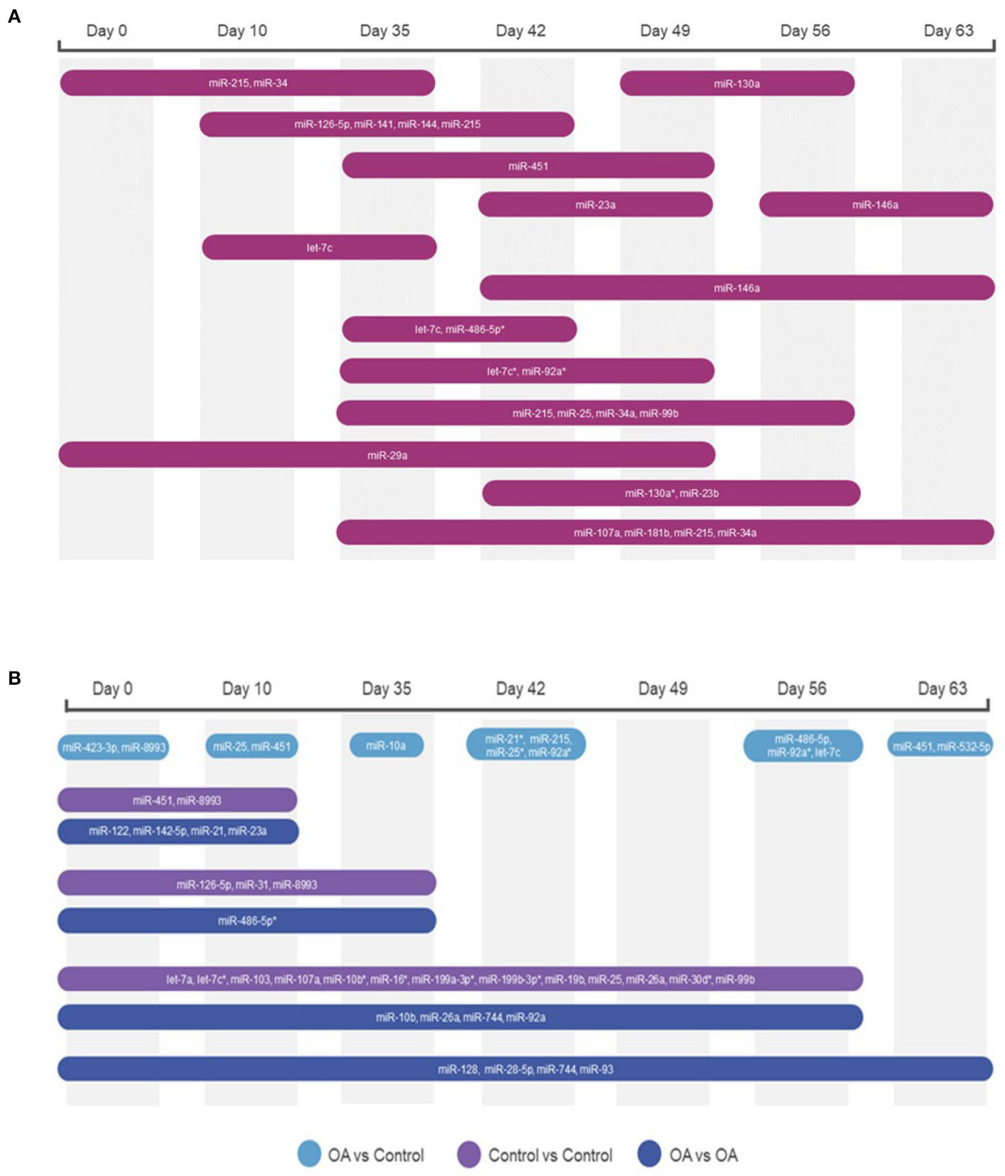
Diagrams summarising the changing miRNA landscape in longitudinal EV samples following small RNA sequencing, miR, eca-miR. **(A)** Plasma-derived EV timeline. **(B)** Synovial fluid-derived EVs for time and diseases. MiRNAs were identified following pairwise comparisons. *p* < 0.05 and * denotes FDR <0.05.

First, we analysed the changes in plasma-derived EV miRNAs before and after the OA induction by comparing day 0 to each subsequent time point (days 10, 35, 42, 49, 56, and 63; Figure 4A; Table 1) and found 16 different DE miRNAs. Following a pairwise statistical analysis of all-time point plasma-derived EVs, we identified 18 DE miRNAs (Table 2A and Figures 4B,C). Five of these were identified in multiple pairwise comparisons (eca-miR-215, eca-miR-34a, eca-let-7c, eca-miR-130a, and eca-miR-146a) (Figure 5). When SF-derived EVs from the control or OA joints were investigated, we identified 31 DE miRNAs (Figures 4D,E and Supplementary material 5). Of these, 20 were altered between OA time points or between control and OA, representing the most likely miRNAs involved in OA pathogenesis (Table 2B and Figure 6). Ten of these were identified in multiple pairwise comparisons (eca-miR-let7c, eca-miR-10b, eca-miR-21, eca-miR-25, eca-miR-26a, eca-miR-451, eca-miR-486-5p, eca-miR-744, eca-miR-8993, and eca-miR-92a; Figure 6). A total of seven miRNAs were DE in the plasma and SF (when differences between control and OA or OA at different time points were accounted for): eca-miR-451, eca-miR-25, eca-miR-215, eca-miR-92a, eca-miR-let-7c, eca-miR-486-5p, and eca-miR-23a. Figure 7 summarises the changing miRNA landscape in longitudinal samples for the plasma (time only; Figure 7A) and SF (time and disease; Figure 7B).

**Table 1:** Differentially expressed miRNAs compared to day 0 in plasma-derived EVs.

**Comparison**	**miRNA**	**logFC**	***p*** **-value**
d0 vs. d10	eca-miR-182	−11.2	0.011
d0 vs. d10	eca-miR-376c	4.3	0.026
d0 vs. d10	eca-miR-144	5.1	0.034
d0 vs. d35	eca-miR-34a	11.4	0.003
d0 vs. d35	eca-miR-215	9.7	0.005
d0 vs. d35	eca-let-7g	−10.3	0.027
d0 vs. d35	eca-miR-107b	−5.6	0.037
d0 vs. d35	eca-miR-151-5p	−6.1	0.047
d0 vs. d42	eca-miR-24	−6.5	0.034
d0 vs. d49	eca-miR-23a	4.0	0.011
d0 vs. d49	eca-miR-1468	9.7	0.018
d0 vs. d49	eca-miR-182	−9.9	0.027
d0 vs. d49	eca-miR-30e	−8.3	0.029
d0 vs. d49	eca-miR-92a	−1.8	0.031
d0 vs. d49	eca-miR-186	−5.7	0.041
d0 vs. d56	eca-let-7g	−6.5	0.031
d0 vs. d56	eca-miR-19b	−5.6	0.031
d0 vs. d56	eca-miR-151-5p	−6.6	0.031
d0 vs. d63	eca-miR-1307	8.8	0.014

*D, day; eca, equine; logFC, log fold change*.

**Table 2A:** Differentially expressed miRNAs following pairwise analysis in plasma-derived EVs.

**Pairwise comparison (day)**	**miRNA**	**logFC**	***p*** **-value**	**FDR**
35 vs. 0	eca-miR-215	7.0	0.023	0.656
35 vs. 0	eca-miR-34a	8.2	0.016	0.656
35 vs. 10	eca-let-7c	4.0	0.047	0.757
42 vs. 10	eca-miR-126-5p	5.0	0.033	0.462
42 vs. 10	eca-miR-141	−9.1	0.005	0.289
42 vs. 10	eca-miR-144	−9.7	0.017	0.462
42 vs. 10	eca-miR-215	−10.1	0.030	0.462
42 vs. 35	eca-let-7c	−2.8	0.037	0.165
42 vs. 35	eca-miR-486-5p	−1.7	0.005	0.048
49 vs. 0	eca-miR-29a	3.1	0.047	0.987
49 vs. 10	eca-miR-451	−3.9	0.019	0.960
49 vs. 35	eca-let-7c	−3.9	0.005	0.050
49 vs. 35	eca-miR-92a	−3.7	0.004	0.050
49 vs. 42	eca-miR-23a	5.4	0.031	0.986
56 vs. 35	eca-miR-215	−7.3	0.034	0.531
56 vs. 35	eca-miR-25	−7.2	0.045	0.531
56 vs. 35	eca-miR-34a	−8.4	0.028	0.531
56 vs. 35	eca-miR-99b	−6.1	0.036	0.531
56 vs. 42	eca-miR-130a	8.8	<0.001	0.021
56 vs. 42	eca-miR-23b	5.1	0.017	0.433
56 vs. 49	eca-miR-130a	8.6	0.006	0.315
63 vs. 35	eca-miR-107a	4.5	0.049	0.549
63 vs. 35	eca-miR-181b	6.3	0.047	0.549
63 vs. 35	eca-miR-215	−8.5	0.013	0.549
63 vs. 35	eca-miR-34a	−7.6	0.033	0.549
63 vs. 42	eca-miR-146a	10.0	0.045	0.983
63 vs. 56	eca-miR-146a	9.6	0.041	0.818

*miRNA, microRNA; eca, equine; logFC, log fold change; FDR, false discovery rate*.

**Table 2B:** Differentially expressed miRNAs following pairwise analysis in synovial fluid-derived EVs.

**Pairwise comparison (day)**	**miRNA**	**logFC**	***p*** **-value**	**FDR**
Control 0 vs. OA 0	eca-miR-423-3p	−4.4	0.044	0.997
Control 0 vs. OA 0	eca-miR-8993	8.5	0.027	0.997
Control 10 vs. Control 0	eca-miR-451	1.5	0.038	0.664
Control 10 vs. Control 0	eca-miR-8993	−10.4	0.015	0.664
Control 10 vs. OA 10	eca-miR-25	1.4	0.025	0.952
Control 10 vs. OA 10	eca-miR-451	1.5	0.022	0.952
Control 35 vs. Control 0	eca-miR-126-5p	−8.3	0.021	0.846
Control 35 vs. Control 0	eca-miR-31	−7.9	0.043	0.846
Control 35 vs. Control 0	eca-miR-8993	−9.4	0.046	0.846
Control 35 vs. OA 35	eca-miR-10a	8.0	0.045	0.917
Control 42 vs. OA 42	eca-miR-21	1.4	0.001	0.034
Control 42 vs. OA 42	eca-miR-215	7.1	0.037	0.313
Control 42 vs. OA 42	eca-miR-25	1.8	0.004	0.050
Control 42 vs. OA 42	eca-miR-92a	1.4	0.003	0.050
Control 56 vs. Control 0	eca-let-7a	1.6	0.037	0.173
Control 56 vs. Control 0	eca-let-7c	2.0	0.000	0.001
Control 56 vs. Control 0	eca-miR-103	−2.8	0.035	0.173
Control 56 vs. Control 0	eca-miR-107a	−2.8	0.034	0.173
Control 56 vs. Control 0	eca-miR-10b	2.0	0.000	0.001
Control 56 vs. Control 0	eca-miR-16	−4.1	0.004	0.045
Control 56 vs. Control 0	eca-miR-199a-3p	1.8	0.001	0.020
Control 56 vs. Control 0	eca-miR-199b-3p	1.8	0.001	0.020
Control 56 vs. Control 0	eca-miR-19b	−5.5	0.011	0.085
Control 56 vs. Control 0	eca-miR-25	−3.4	0.034	0.173
Control 56 vs. Control 0	eca-miR-26a	1.3	0.006	0.049
Control 56 vs. Control 0	eca-miR-30d	2.3	0.004	0.046
Control 56 vs. Control 0	eca-miR-99b	3.3	0.030	0.173
Control 56 vs. OA 56	eca-let-7c	0.5	0.022	0.074
Control 56 vs. OA 56	eca-miR-486-5p	0.9	0.015	0.074
Control 56 vs. OA 56	eca-miR-92a	1.6	0.005	0.046
Control 63 vs. OA 63	eca-miR-451	1.6	0.022	0.993
Control 63 vs. OA 63	eca-miR-532-5p	6.2	0.035	0.993
OA 10 vs. OA 0	eca-miR-122	9.4	0.035	0.762
OA 10 vs. OA 0	eca-miR-142-5p	9.1	0.019	0.762
OA 10 vs. OA 0	eca-miR-21	1.4	0.027	0.762
OA 10 vs. OA 0	eca-miR-23a	4.2	0.007	0.588
OA 35 vs. OA 0	eca-miR-486-5p	−1.9	0.008	0.046
OA 56 vs. OA 0	eca-miR-10b	1.0	0.046	0.991
OA 56 vs. OA 0	eca-miR-26a	0.9	0.046	0.991
OA 56 vs. OA 0	eca-miR-744	10.0	0.032	0.991
OA 56 vs. OA 0	eca-miR-92a	−1.6	0.037	0.991
OA 63 vs. OA 0	eca-miR-128	7.3	0.028	0.703
OA 63 vs. OA 0	eca-miR-28-5p	8.8	0.036	0.703
OA 63 vs. OA 0	eca-miR-744	8.5	0.020	0.703
OA 63 vs. OA 0	eca-miR-93	7.5	0.023	0.703

*OA, osteoarthritis; miRNA, microRNA; eca, equine; logFC, log fold change; FDR, false discovery rate*.

#### snoRNAs

**Figure 8:**
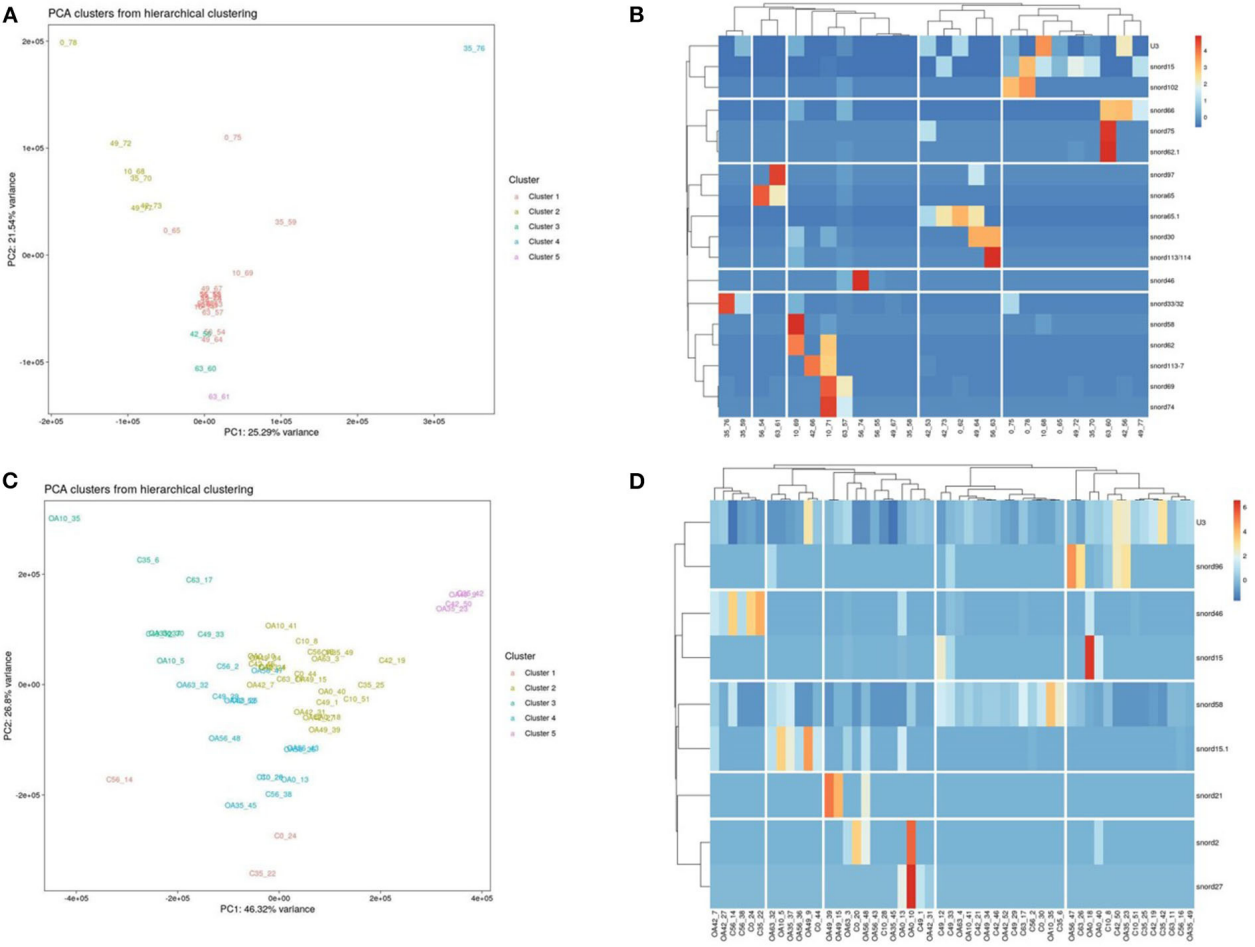
Differentially expressed (DE) snoRNAs in plasma and synovial fluid-derived EVs. **(A)** PCA of DE snoRNAs for plasma-derived EVs. **(B)** Heatmap of DE snoRNAs (*p* < 0.05) in plasma-derived EVs. **(C)** PCA of snoRNAs for synovial fluid-derived EVs (C, control; OA, osteoarthritis). Numbering of samples: the first number is the day of sampling (0, 10, 35, 42, 49, 56, and 63). The second number is the original sample ID, *n* = 4 horses. **(D)** Heatmap of DE snoRNAs (*p* < 0.05) in synovial fluid-derived EVs. Heatmaps were created using the R pheatmap package. Clustering was conducted with the “average” method and by correlation distancing. Scaling is unit variance scaled expression and based on read counts. Clustering distances are based on Pearson correlation.

Following a pairwise statistical analysis considering the donor effect of plasma-derived EVs, we identified 16 DE snoRNAs (Table 3A and Figures 8A,B). Ten of these were identified in multiple pairwise comparisons (snord102, U3, snord113, snord113/114, snord15, snord66, snord69, snord58, snord62, and snord65). When SF-derived EVs from the control or OA joints were investigated, we identified eight DE snoRNAs (Table 3B and Figures 8C,D). Six of these were identified in multiple pairwise comparisons (U3, snord27, snord15, snord46, snord27, and snord58). There were four DE snoRNAs in the plasma and SF (U3, snord15, snord46, and snord58).

**Table 3A:** Differentially expressed snoRNAs following pairwise analysis in plasma-derived EVs.

**Pairwise comparison (day)**	**snoRNA**	**logFC**	***p*** **-value**	**FDR**
56 vs. 0	U3	−6.3	<0.001	<0.001
56 vs. 0	snord102	−7.8	<0.001	0.002
56 vs. 0	snord46	4.6	0.001	0.003
49 vs. 42	snord113-7	−4.2	0.003	0.041
56 vs. 49	snord15	−6.7	0.032	0.097
63 vs. 56	snord66	7.4	0.009	0.092
49 vs. 0	U3	−7.0	0.006	0.082
56 vs. 0	snord15	−7.9	0.032	0.096
56 vs. 0	snord102	−8.2	0.048	0.096
63 vs. 0	snord66	9.8	0.024	0.094
35 vs. 10	snord69	−9.9	0.003	0.046
42 vs. 10	snord113/114	−3.9	0.005	0.076
42 vs. 10	snord69	−8.2	0.013	0.076
42 vs. 10	snord58	−6.9	0.015	0.076
42 vs. 10	snord62	−7.1	0.016	0.076
42 vs. 10	snord30	−8.0	0.025	0.095
56 vs. 10	snord69	−11.0	0.002	0.026
56 vs. 10	snord15	−7.1	0.013	0.092
56 vs. 10	U3	−6.8	0.027	0.092
56 vs. 10	snord62	−7.1	0.031	0.092
56 vs. 10	snord58	−7.0	0.034	0.092
56 vs. 10	snord74	−8.7	0.039	0.092
56 vs. 35	snord33/32	−9.3	0.006	0.063
56 vs. 35	U3	−8.8	0.019	0.094
63 vs. 35	snord66	11.0	0.012	0.096
63 vs. 35	snord97	11.0	0.012	0.096
63 vs. 35	snord75	10.0	0.019	0.096
63 vs. 35	snord113/114	9.3	0.039	0.096
63 vs. 35	snord69	9.2	0.039	0.096
63 vs. 35	snord62	9.0	0.039	0.096
63 vs. 35	snora65	8.0	0.039	0.096
56 vs. 42	U3	−7.7	0.011	0.080
56 vs. 42	snora65	−8.4	0.028	0.099
56 vs. 49	snord15	−7.8	0.029	0.086

*snoRNA, small nucleolar RNA; logFC, log fold change; FDR, false discovery rate*.

**Table 3B:** Differentially expressed snoRNAs following pairwise analysis in synovial fluid-derived EVs.

**Pairwise comparison (day)**	**snoRNA**	**logFC**	***p*** **-value**	**FDR**
Control 35 vs. Control 0	U3	−0.4	0.027	0.082
OA 35 vs. OA 0	snord27	−9.9	0.038	0.094
OA 35 vs. OA 0	snord2	−9.0	0.043	0.094
OA 35 vs. OA 0	snord46	−9.5	0.043	0.094
OA 35 vs. OA 0	snord15	−9.5	0.047	0.094
OA 49 vs. OA 0	snord27	−9.8	0.023	0.095
OA 49 vs. OA 0	snord46	−9.4	0.036	0.095
OA 49 vs. OA 0	snord21	9.3	0.047	0.095
OA 56 vs. OA 0	snord46	−8.9	0.043	0.093
OA 56 vs. OA 0	snord15	−8.8	0.048	0.093
OA 56 vs. OA 0	snord27	−9.2	0.052	0.093
OA 56 vs. OA 0	U3	−1.3	0.053	0.093
Control 56 v OA 56	snord46	8.8	0.024	0.098
Control 63 vs. Control 0	snord58	7.0	0.003	0.034
OA 10 vs. OA 0	snord58	2.3	0.019	0.097
OA 10 vs. OA 0	snord46	−3.5	0.040	0.099
OA 10 vs. OA 0	snord15	3.0	0.060	0.100
Control 49 vs. OA 49	snord58	4.4	0.009	0.094
Control 56 vs. OA 56	snord15	−2.9	0.000	<0.001
Control 56 vs. OA 56	snord96	−2.8	0.001	0.010
Control 63 vs. OA 63	snord46	−3.7	0.003	0.043

*snoRNA, small nucleolar RNA; logFC, log fold change; FDR, false discovery rate*.

#### Other non-coding RNAs

Pairwise comparisons for other non-coding RNAs considering the donor effect were conducted on the plasma and SF. In the plasma, we found five tRNAs, eight lncRNAs, and five snRNAs DE (FDR <0.1) Additionally, in the SF, we identified five tRNAs, two lncRNAs, and four snRNAs DE (FDR <0.1; Supplementary material 6).

### Bioinformatics analysis of DE miRNAs in plasma or SF

#### Plasma pairwise comparison

**Figure 9:**
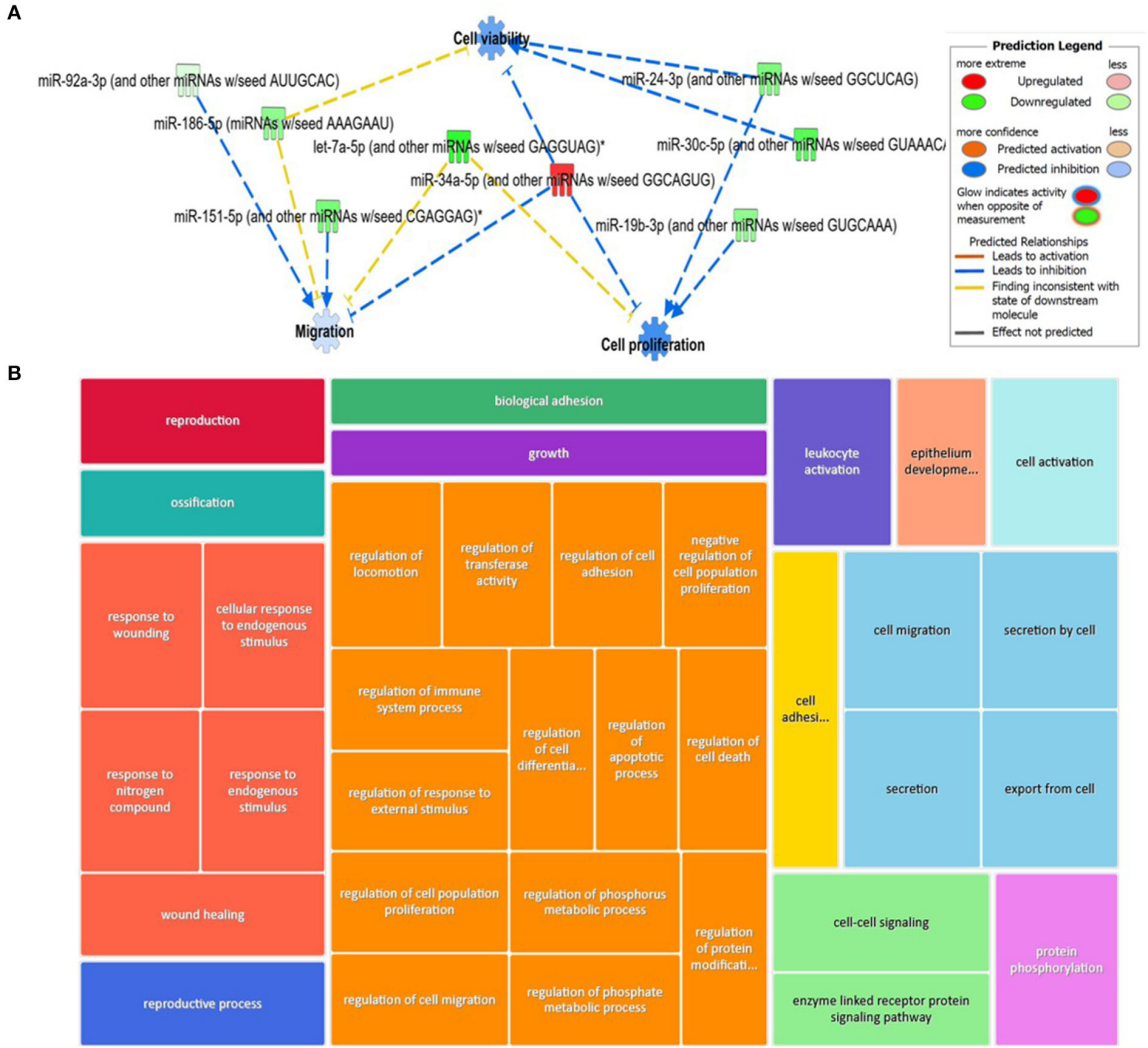
Bioinformatics analysis of the differentially expressed (DE) plasma-derived EV miRNAs following small RNA sequencing and their putative mRNA targets. **(A)** IPA of the 27 DE miRNAs following all pairwise comparisons compared to day 0 showed that cell viability, proliferation, and migrations were activated. **(B)** Treemap of the top 100 GO terms. Hierarchical level is represented by a different coloured rectangle (branch), each containing smaller rectangles (leaves). The size of the space inside each rectangle is based on the measured value. GO biological processes associated with dysregulated miRNA targets were identified following the TargetScan philtre module in IPA. ToppGene was used to perform a functional enrichment analysis of predicted miRNA targets to highlight biological processes most significantly affected by dysregulated miRNA-mRNA interactions. GO terms (FDR <0.05) were summarised and visualised using Revigo and Cytoscape. The allowed similarity setting in Revigo is medium.

For the plasma-derived EVs, we first looked at the set of 16 DE miRNAs following pairwise comparisons compared to day 0. IPA identified the top diseases and functions with negative activated Z-scores (used to infer the activation states of biological functions based on comparison with a model that assigns random regulation directions; for negative, this is related to prediction of inhibition) as migration (*p* = 9.14 × 10^−4^), cell proliferation (*p* = 5.32 × 10^−5^), and cell viability (*p* = 8.7 × 10^−2^; Figure 9A). To investigate the position of the 27 (22 when duplicates were removed) DE miRNA plasma expression networks, we then determined their putative target genes using IPA Target Philtre, which integrates computational algorithms with multiple miRNA databases. We used a threshold of “experimentally validated” or “highly predicted.” This matched the miRNAs to 920 mRNA targets. The mRNA targets were input into the gene ontology tool ToppGene; then, 1,626 biological processes identified were visualised using Revigo and Cytoscape (Figure 9B). The top 100 biological processes were visualised in Treemap (Figure 9B).

#### SF pairwise comparisons

**Figure 10:**
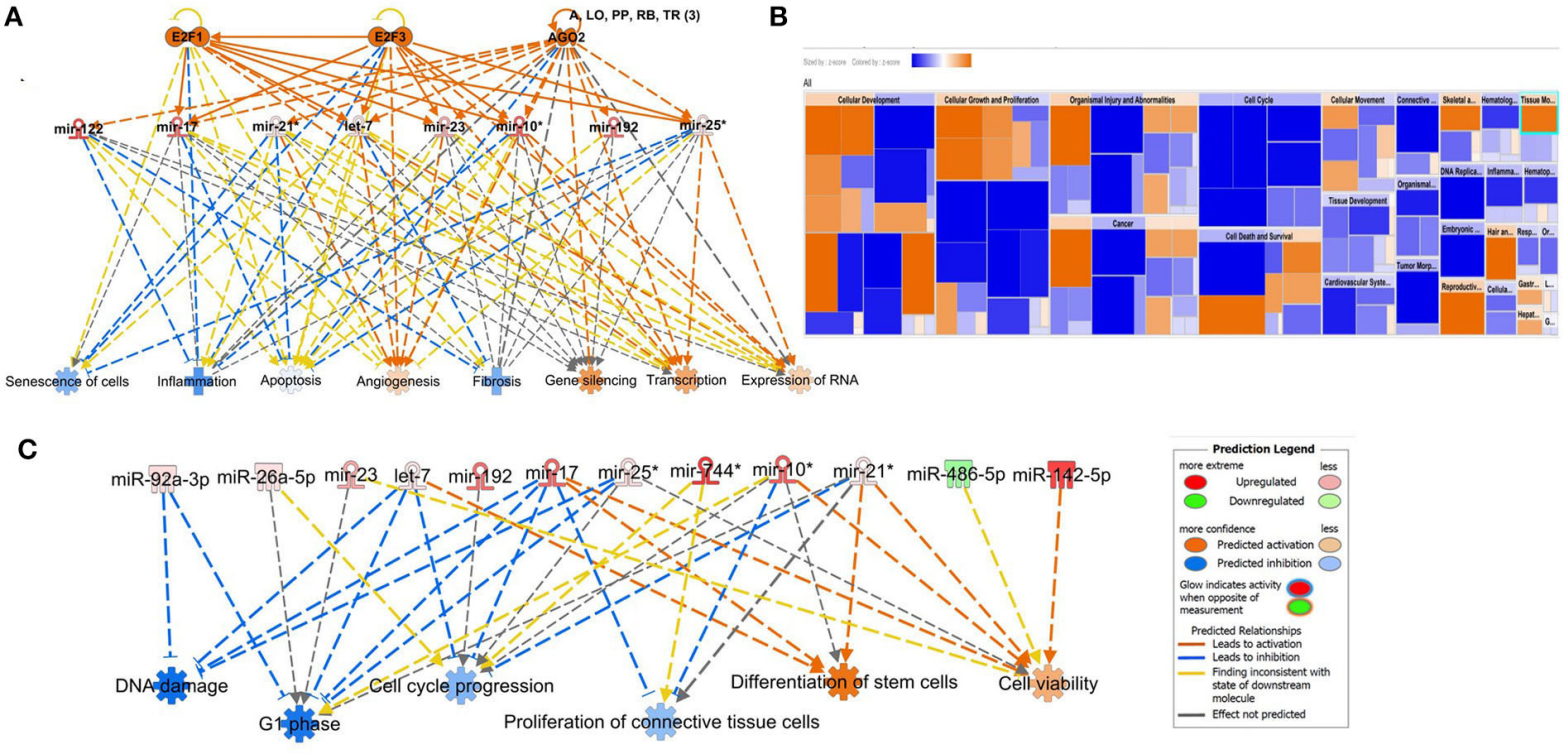
Pathway analysis of differentially expressed (DE) miRNAs in synovial fluid following small RNA sequencing using IPA. **(A)** Regulatory effect network for E2F1, E2F3, and AGO2 action, senescence, inflammation, apoptosis, angiogenesis, fibrosis, gene silencing, transcription, and expression of RNA *via* differentially expressed miRNAs. **(B)** Heatmap sized by the *Z*-score of biological processes with a scale where blue is inhibited and orange is activated. **(C)** Significant diseases and functions based on *Z*-score associated with DE miRNAs. Figures are graphical representations of molecules identified in our data in their respective networks. Red nodes are upregulated and green nodes are downregulated gene expression. Intensity of colour is related to higher fold-change. Legends for the main features in the networks are shown. Biological function is dependent on whether it is predicted to be activated or inhibited.

We input for bioinformatics analysis only DE miRNAs in SF pairwise comparisons altered between control (sham) and OA or between OA samples at different time points. Thus, 27 miRNAs were input into IPA “Core Analysis” (Supplementary material 7). E2F Transcription Factors 1 (*p* = 7.37 × 10^−7^) and 3 (*p* = 3.68 × 10^−9^) and Argonaute RISC Catalytic Component 2 (*p* = 3.75 × 10^−17^), an initiator of target mRNA degradation, were significant upstream regulators of eight DE miRNAs with downstream effects on senescence (*p* = 3.66 × 10^−4^), inflammation (*p* = 2.32 × 10^−3^), apoptosis (*p* = 7.57 × 10^−4^), angiogenesis (*p* = 1.17 × 10^−5^), fibrosis (*p* = 6.84 × 10^−16^), gene silencing (*p* = 1.74 × 10^−10^), transcription (*p* = 0.002), and expression of RNA (*p* = 0.003; Figure 10A). A heatmap derived from IPA identified biological processes based on activation *Z*-score (Figure 10B). This highlighted that cell cycle was predicted to be inhibited (negative activation *Z*-score) and contributed to by G1 phase (*p* = 1.55 × 10^−8^, *Z*-score −1.6), G1/S phase transition (*p* = 3.52 × 10^−6^, *Z*-score −1.6), interphase (*p* = 6 × 10^−8^, *Z*-score −1.5), senescence (*p* = 3.7 × 10^−4^, *Z*-score −0.67), and cell cycle progression (*p* = 1.5 × 10^−5^, *Z*-score −0.66; Figure 10C and Supplementary material 7). Furthermore, whilst DNA damage (*p* = 1.32 × 10^−4^, *Z*-score −2) and cell proliferation (*p* = 9.06 × 10^−7^, *Z*-score −2.2) were also reduced, cell viability (*p* = 1.6 × 10^−3^, *Z*-score 0.75) and differentiation of stem cells (*p* = 2.36 × 10^−4^, *Z*-score 1.03) were predicted to be increased (Figure 10C).

## Discussion

Fundamental studies that contribute to characterisation and understanding of early OA will serve to elucidate disease pathogenesis and identify novel molecules as biomarkers for early detection or that act as therapeutic targets. The demonstration that EVs are a method of cell communication has caused a recent increase in publications focused on EVs (56, 57). In this study we have, for the first time, studied the sncRNA EV content temporally in OA using an equine model. In our previous study, we have confirmed the development of OA in our equine carpal osteochondral fragment model by histological evaluation. There was significant cartilage degradation and synovial membrane inflammation in OA joints (54).

Similarities in the pathogenesis and clinical signs of OA in humans and horses are leading the expansion of equine translational studies (58, 59), and with the advantages of histological and anatomical similarities to human joints, it is easy to conduct longitudinal sampling of SF and blood in the animal model. However, the use of a horse as a model is limited by high costs and ethical considerations and reduces the number of animals in studies. In our experiment, we had access to samples from four horses and were able to examine, for the first-time in any species, temporal OA changes in EV characteristics and sncRNA cargo using SF and plasma and spanning seven time points. Whilst our groups have examined the sncRNA cargo in equine SF in early OA (8) and the temporal changes in miRNAs in SF from the same equine osteochondral fragment model (36), here, using less time points, we tried to identify the changing sncRNA landscape in SF or plasma-derived EVs.

It is challenging for most experimental designs to discriminate exosomes from microvesicles in terms of their size, cargo, properties, and origin (60). Consequently, most research represents the characteristics of a heterogenous group of nanovesicles, which are derived from both endosomal multivesicular bodies and plasma membrane budding. In our study, we used both NTA and interferometric images (Exoview) using tetraspanin antibodies to size and count individual EVs. First, we conducted NTA to characterise the EV profile in all the samples of SF and plasma from the model. We then used pooled the samples at selected time points for ExoView to financial constraints. To isolate our EVs, we used carried out exclusion chromatography to separate EVs from proteins and lipids as we had only small volumes of our biological fluids. Others have recommended this isolation method when there are limited volumes of plasma and SF, and downstream analysis includes sncRNA and protein cargo (58, 59). Ultracentrifugation alone of SF-derived EVs increased contamination and aggregation, whilst size exclusion chromatography increased EV enrichment (61).

EVs are involved in multiple functional roles in joint homeostasis [reviewed (62)]. The EV characteristics of size and concentration can be affected by any external stimulus, which significantly alters the formation, release, and uptake of EVs (63). By NTA, we measured overall sizes and quantity of isolatable EVs. This method enables acceptable repeatability and reproducibility (64). However, the results must be interpreted considering inherent biological sample diversity. The samples contained a heterogenous group of EVs of various sizes and are comparable only with a caution to preparations from cell culture and other plasma or SF studies as results are also dependent on methods of EV isolation. Having utilised the size exclusion chromatography method, our measurements displayed minimal levels of background noise reflecting the reduction of protein aggregates or cellular components compared to ultracentrifugation isolation methods (59). Our NTA size estimates were within EV range and similar to other plasma (65) and SF (61, 66) studies using the size exclusion chromatography method. However, compared to a recent plasma EV study on human OA using EV precipitation and nanoparticle tracking methods, the equine plasma EVs were smaller (human 235 nm, equine 158 nm) (24), which may be because of the isolation methods used. Comparing both biofluids, the measured particle sizes were overall greater in the SF than in the plasma. We did not find any difference in EV concentration, size, mode, D10, D50, or D90 in the SF temporally or with OA induction in the model. However, mean plasma EV size was found to be decreased on day 49, and D90 was decreased on day 63. Interestingly there are few studies characterising the size and concentration of EVs in SF or plasma of normal and OA samples. Our results concur with one study in which NTA was performed to characterise EVs in normal human and OA SFs (66). However, it should be noted that a precipitation exosome kit was used in this study for EV isolation. Following exosome isolation from human knee SF, there was increase in exosome concentration from early to severe knee OA without changes in the average particle size (67). In a further study on early and late stage knee OA following isolation of exosomes by ultracentrifugation of biofluids, whilst for plasma there was no difference in concentration of exosomes, in SF the expression of exosomes in early OA and late-stage OA was higher than that in controls (68).

As NTA does not specifically measure EVs but also co-isolates contaminants, we also used Exoview™ to characterise SF and plasma-derived EVs at selected time points. This technology is used to conduct single particle interferometric reflectance imaging sensing together with antibody-based chip capture and fluorescence detection that enables single vesicle identification. Additionally, it has a lower size limit of detection than scattering based techniques (69). We had previously tested EVs isolated from equine SF and plasma on both mouse and human tetraspanin chips and found that the human chips captured EVs more efficiently (data not shown). Whilst classical EV markers are CD9, CD63, and CD81, some cells secrete EVs devoid of CD63 (70) and so a panel of antibodies is preferable to confirm EV isolation. There was minimal staining for CD63 indicating either poor antibody sequence homology or rareness of the EVs. Unfortunately, we could not cheque for sequence homology because of proprietary constraints; therefore, some cautions should be used in not over-interpreting the results from antibody characterisation as we do not know the actual cross reactivity. However, for CD9, CD81, and CD9, protein sequence homology is 92, 95, and 84%, respectively. As we suspected a lack of sequence homology in our preliminary study, we did not analyse the CD63 results further. Because of prohibitive costs, we only ran a subset of the SF and plasma samples on the Exoview platform. In the plasma, there was an apparent change in size following the OA induction. In the SF, no temporal or OA-related change was evident for CD9-positive EVs; however, for CD81-positive EVs, there was. CD81 expression changed with time in the SF. The differences in tetraspanin expression may indicate that the EVs present in the SF and plasma could have different functional activities, as tetraspanins are important for functionalities of EVs as their function is dependent on their ability to interact with target cells. These, in turn, are determined by surface receptors existing in each EV subtype (71). Tetraspanin complexes are EV surface receptors that would define target cells to bind (72). A study on seminal plasma demonstrated an association of CD63 with CD9 and CD81 in exosomes, indicating a possible synergistic effect of these tetraspanins (73). Simultaneous immunostaining enabled dual immunofluorescent analysis, and therefore co-localisation of CD9 and CD81 was assessed. In the plasma on days 0 and 42, there were similar co-localisation profiles, but the proportion of double-positive EVs was reduced significantly on day 63. This could potentially indicate a switch in tetraspanin phenotype in OA. The biological implications of this switch are unknown. One limitation of our study, which should be noted with respect to Exoview data, is the limited number of samples analysed; therefore, further studies are necessary before hard conclusions can be drawn.

Since the identification of RNA in EVs (74), there has been a rise in the interest of using EV-RNAs as biomarkers (75). Others have demonstrated that the relative concentrations of miRNAs secreted in EVs differs from those found in the cell, or serum/plasma concentrations of miRNAs (76). Because of the role of EVs in cell communication, we hypothesised that sncRNA changes due to OA may be found in both SF and plasma EVs. SF-derived EVs are likely to come from a number of sources including cartilage, subchondral bone, synovium, and the circulation. We used a non-biassed approach, small RNA sequencing, to remove bias, and whilst most blood (21, 23, 77–79) or SF studies (21, 22) on OA have looked at miRNAs alone, we investigated a number of non-coding RNAs in our samples. To our knowledge, this is only the second study to investigate EVs derived from plasma in OA (24) and the first to describe SF-derived EV sncRNAs in OA.

To reduce contamination with residual platelets, all the plasma samples were centrifuged within 30 min after blood collection at 3,000 × *g* 15 min as previously recommended (80). Whilst we cannot discriminate contamination by miRs included in platelets in our plasma samples, this methodological step should have reduced it to a minimum. Future work could investigate platelet-derived EV markers such as CD31, CD41, CD42a (81) in order to determine the level of potential platelet contamination, or use flow cytometry and antibodies to remove potential platelet-derived EVs. We used EDTA collection tubes. miR content is different among plasma EVs, serum, and plasma (78). Care should be taken when comparing results not only between plasma and serum in similar studies but also in plasma studies depending on the collection tube used. Serum and citrate plasma contain increased numbers of platelet-derived EVs compared to acid citrate dextrose and EDTA plasma samples in one study (82).

In the plasma, we found 16 different DE miRNAs between day 0 and other time points and 22 DE after all the pairwise comparisons were conducted. We hypothesise that changes in the plasma could, in the initial few weeks, be due to OA and sham joint changes but would during the later stages of the model be due to OA changes alone. Of the miRNAs DE some have been found to change in OA in other studies; has-miR-126, has-miR-29 (23), and has-miR-146a in four human studies (21, 23, 78, 83). Interestingly, whilst one study on plasma found no DE miRNAs in plasma-derived EVs in OA, miR-146a was one of the most highly expressed miRNAs identified (24). In our study, we demonstrated that eca-miR-146a was significantly increased between days 42 and 63 and 56 and 63. Serum has-miR-146a-5p was significantly increased in women with knee OA compared with controls (74), and has-mir-146a has a vital role in maintaining cartilage homeostasis and increases in cartilage in early OA (84, 85). Furthermore, in a mouse study, surgically induced OA treated with a miR-146a inhibitor significantly alleviated cartilage destruction by targeting Camk2d and Ppp3r2 (84). Thus, others have identified an increase in miR-146 in human serum in OA, whilst we identified that it was also increased in plasma-derived EVs following OA induction. This seems an across species effect of cartilage degradation in OA, which can then be evident in blood and blood-derived EVs. The increase in plasma EV miR146 could be derived from OA joints. Further studies are necessary to determine if plasma EV-derived miR-146 is an effect of cartilage destruction and could be targeted to treat OA.

In the SF-derived EVs, we identified 31 miRNAs that were DE between the control and OA or temporally in OA. Some have been previously identified as DE in other studies, including eca-let7c, eca-miR-10a, eca-miR-122, eca-miR-215, and eca-miR-23a, in SF derived from horses 28 days after the osteochondral model was commenced in the original study containing a larger cohort of nine horses (54).

Seven miRNAs were DE in the plasma and SF-derived EVs when the data were analysed between different time points; eca-miR-451, eca-miR-25, eca-miR-215, eca-miR-92a, eca-miR-let-7c, eca-miR-486-5p, and eca-miR-23a. Thus, these miRNAs represent the most promising biomarkers for further studies. An ideal biomarker for early equine OA would be sourced from blood rather than SF, but the fact that changes occur in both biofluids suggests that they are associated with OA changes in our model. Interestingly, eca-miR-215, eca-let7-c, and eca-miR-23a were also identified in our study in which we isolated sncRNAs from SF. They were altered on day 28 compared to day 0 in the same model and the same horses (54). Eca-miR-23a was also increased in equine early OA SF (8) and increased in late vs. early human OA SF (22). Has-mir-23a contributes to OA progression by directly targeting SMAD3 (86). Generally, our miRNA findings support our other study with this model in which we found that OA disease progression caused early changes in SF miRNA expression patterns on day 28 (24).

We can only compare the plasma-derived EV results with one previous non-temporal study in OA in which no DE miRNAs were identified. This study used 23 OA patients and 23 controls and isolated EVs by precipitation followed by sequencing the samples on a sequencing platform similar to that used in this study (24). These discrepancies may be related to differences in species, sample type, disease stage or severity, methodology, gender differences, and other factors among studies.

EVs are vehicles for exchange of bioactive signalling molecules in cartilage and between joint tissues to promote joint homeostasis and arthritis pathogenesis. SncRNAs in condition-specific EVs may function as a unique set of non-coding RNAs. We know that miRNAs in particular act as regulatory molecules, but that other sncRNAs may also act as components of EVs contributing to OA pathogenesis by mediating cell-cell and tissue-tissue communication in osteoarthritic joints. Understanding the role of plasma-derived EVs in OA pathogenesis needs further studies, which we are currently conducting. In order to understand the potential role of the panel of miRNAs changing in our model, we conducted a pathway analysis of the DE miRNAs. First, we took DE plasma-derived miRNAs following pairwise comparisons compared to day 0. This was to define changes at a pathway level temporally compared to prior to OA induction (day 0). A “Core Analysis” of the miRNAs in IPA described a predicted inhibition of migration, cell proliferation, and cell viability. The carpal osteochondral fragment model specifically simulates a post-traumatic OA phenotype. Therefore, every event from carpal chipping to erosion of cartilage is considered part of the pathogenesis of the disease. This suggests that plasma-derived miRNA EV cargo changing in the model would inhibit cell migration, proliferation, and viability. However, the origin of these EVs cannot be confirmed but, if related to the model, would represent changes occurring in both the OA and sham joint processes including wound healing and repair, bone, cartilage, and synovial damage, and reparative processes of these tissues plus inflammation. In an OA mesenchymal stem, cells migrate and proliferate into the damaged cartilage area (87), and inflammatory cells can migrate to the joint (88). By reducing migration, proliferation and viability, the plasma miRNA EV cargo could potentially affect the interplay of various cells in the OA joint.

Next, we took the 22 DE miRNA plasma expression network, used IPA to predict their target genes, and then condensed the list of biological pathways affected by these mRNAs. This identified many terms related to cell response (including would healing and endogenous stimuli), regulation of cellular processes (including cell death, proliferation, apoptosis, migration, and metabolic processes), and cell signalling, secretion, and activation. As the EV-miRNA cargo was from the plasma, it is difficult to distinguish if these effects are due to OA changes alone or arthroscopy of both the OA and sham joints. Despite this, our findings potentially offer an insight into early changes in post-traumatic OA. However, by investigating the potential pathways of DE miRNAs in the SF (which we hypothesise would be more likely to be due to disease induction), we detailed the potential role of this cargo and its effects in early post traumatic OA in the joint. Interestingly, whilst one would expect the Argonaute RISC catalytic component to be an upstream regulator of miRNAs [as the Argonaute family of proteins has a role in RNA interference (89)], E2F Transcription Factors 1 and 3 were also predicted. This has a role in accelerating cell proliferation and promoting inflammation signalling (90), and E2F1 targets may be affected in OA pathogenesis (91).

Additionally, we analysed the expression changes of other sncRNAs. To our knowledge, for the first time in an OA model, we have identified changing plasma and SF-derived EV tRNAs, lncRNAs, and snRNAs. These could be important molecules for understanding and treating OA. For example, Liu et al. showed that exosomes derived from human mesenchymal stem cells contained lncRNA KLF3-AS1 and promoted chondrocyte proliferation *in vitro*, whilst in an *in vivo* collagenase-induced OA model, these exosomes improved cartilage repair (92).

We also investigated the changes in snoRNAs. From our previous study, we have known that snoRNAs have an important role in joint homeostasis (93), mouse joint ageing (17), chondrogenic differentiation (94), cartilage ageing (15), OA (6, 7), and early OA SF (8). Interestingly, in the plasma, we found that 82% of DE snoRNAs in the plasma were also identified in ageing/OA human cartilage study (7), including Snord113/114, snord15, snord30, snord32/33, snord46, snord58, snord62, snord66, snord69, snord74, snord75, and U3. We have also shown snord113 to be DE in ageing mouse joints (17), ageing equine chondrocytes (95), ageing equine cartilage (96); U3 to be DE in ageing mouse serum (17), OA cartilage and chondrocytes (6); snord32/33 to be DE in ageing mouse serum (17) and OA human and equine cartilages where it has a role in oxidative stress (97). For DE snoRNAs the in synovial fluid, 88% of DE snoRNAs in the plasma were also identified in an ageing/OA human cartilage study (7) including snord15, snord2, snord21, snord46, snord58, snord96A, and U3. Furthermore, snord96A was DE in equine SF in early OA (8) and snord58 in ageing human cartilage (unpublished work). Four snoRNAs, U3, snord15, snord46, and snord58, were found to be DE in both plasma and SF-derived EVs and were all altered in pairwise comparisons in OA. Changes in U3 are particularly interesting given our knowledge of its role in OA and impact on protein translational apparatus (6). This snoRNA was reduced in the plasma at multiple time points and in the SF between days 0 and 56. It is essential for rRNA maturation, acting as a spliceosome during ribosome biogenesis and releasing 18S rRNA from the precursor 47S rRNA (98). Impairment of 18S rRNA is suggested to further decrease levels of 5.8S and 28S rRNA in experimental U3 impaired human articular chondrocytes (6), whilst in OA human articular chondrocytes following U3 downregulation, there was a reduction in the expression of chondrogenic genes accompanied by increased levels of chondrocyte hypertrophy genes. OA-related U3 downregulation affects the protein translation capacity of chondrocytes *in vitro* (6). Additionally, sequences of U3 have been suggested to have miRNA-like abilities, accomplishing effective RNA-silencing *in vitro* in at least two human cell lines (98). Overall, the changes in snoRNAs in EVs in both the plasma and the SF provide an insight into their role in the pathogenesis of OA, as traditionally OA has been defined as an imbalance between joint anabolism and catabolism. With our increasing evidence that it is an acquired ribosomopathy (93), there is a potential role for EV-derived snoRNAs to contribute to OA pathogenesis *via* its cell communication role. We are currently studying sncRNA cross-talk in joint cells to shed more light on this hypothesis (99). Thus, EV snoRNAs may provide novel molecular opportunities for the development of OA therapeutics.

We realise that there are a number of limitations in the study. The plasma and SF samples used in this study were stored at −80°C for 2 years prior to analysis, and we were limited in the volumes of plasma and SF we had because the samples were used for multiple platforms. Although collection, handling, and storage were the same for all the samples, any inconsistencies in these procedures may alter the levels of sncRNAs. We used spike-ins throughout the sequencing methodology to increase reproducibility, and indeed the number of sncRNAs was similar to those observed by others in plasma-derived EVs (24), suggesting that our results are likely to be representative for sncRNAs in SF and plasma EVs. The age range of the donors was 2.5–7 years old. Therefore, we cannot discount possible age-related changes in the EV characteristics or sncRNA expression identified in this study. We have previously described that mouse serum miR expression in young (8 months) vs. old (18 months) mice changes (18). Our laboratory found no changes in SF-derived EV characteristics or selected miR expression between young (0–5 years) and old (14–21 years) horses (100).

Unfortunately, we were unable to validate our findings in the same samples by qRT-PCR as there was not enough remaining sample for EV extraction.

## Conclusion

Sequencing of temporal samples for sncRNAs in an equine model produced unbiased profiling of the circulating and SF-derived EV sncRNAome and identified a unique panel of DE sncRNAs during initiation and progression of early post-traumatic equine OA. We characterised plasma and SF-derived EVs in equine OA for the first time and demonstrated that differences in tetraspanin expression may indicate that in early OA they could represent changing functionalities of EVs. The seven DE miRNAs in plasma and SF-derived EVs, eca-miR-451, eca-miR-25, eca-miR-215, eca-miR-92a, eca-miR-let-7c, eca-miR-486-5p, and eca-miR-23a, and four snoRNAs, U3, snord15, snord46, and snord58, identified temporally between specific time points represent exciting molecules for future studies.

## Data availability statement

The NGS data presented in this study are available using GEO ID GSE200330.

## Ethics statement

The animal study was reviewed and approved by the Danish Animal Experiments Inspectorate (permit 2017-15-0201-01314) and Local Ethical Committee of the Large Animal Teaching Hospital of University of Copenhagen approved the experimental protocol. All procedures were undertaken according to EU Directive 2010/63/EU for animal experiments.

## Author contributions

MW, LB, and SJ: conception and design of the animal experiment. JA, MP, EC, MW, LB, and SJ: collection of data. JA, MP, EC, AD, MH, VJ, MW, and SJ: analysis and interpretation of data. JA, MP, AD, and MH: statistical analysis and expertise. MP and JA: drafting of the article. SJ and MP: obtaining of funding. All authors: critical revision of the article for important intellectual content and final approval of the article.

## Funding

This study and JA were funded by the Horserace Betting Levy Board. MP was funded by Wellcome Trust Clinical Intermediate Fellowship (grant 107471/Z/15/Z). LB and the animal experiment were funded by the Independent Research Fund Denmark, Technology and Production Sciences (grant number DFF - 7017-00066, 2017) and Gerda and AageHaensch's Foundation. MW was funded by a Ph.D. scholarship jointly awarded by the University of Copenhagen, the Technical University of Denmark, and the Swedish University of Agricultural Science.

## Conflict of interest

Authors AD and MH were employed by TAmiRNA GmbH. The remaining authors declare that the research was conducted in the absence of any commercial or financial relationships that could be construed as a potential conflict of interest.

## Publisher's note

All claims expressed in this article are solely those of the authors and do not necessarily represent those of their affiliated organisations, or those of the publisher, the editors and the reviewers. Any product that may be evaluated in this article, or claim that may be made by its manufacturer, is not guaranteed or endorsed by the publisher.
