# Supplementary material for: Small non-coding RNA landscape of extracellular vesicles from a post-traumatic model of equine osteoarthritis
Source: Front Vet Sci. 2022 Aug 8;9:901269. doi: 10.3389/fvets.2022.901269 (PMC9393553; doi:10.3389/fvets.2022.901269)
Supplement: Supplementary file 1 [file Table_1.DOCX]

Supplementary File 1. Representative gross and histological images of carpus joints with and without OA induction. A. Gross photograph of A; control joint and B; joint following OA induction taken at sacrifice at Day 70 from the same horse showing mild to moderate changes. C. A representative histological image of a horse following OA induction. OA was successfully induced in middle carpal joint. A. Articular cartilage from the control joint (score 9/20), D. Articular cartilage from the third carpal bone in the OA joint (score 13/20) from the same horse. Hematoxylin and eosin staining 70 days after surgical induction of OA. Note the marked chondrocyte necrosis and focal cell loss in the OA joint. Furthermore, in this particular cartilage section of the OA joint the cartilage is lost and replaced by fibrillating fibrocartilage.

A.


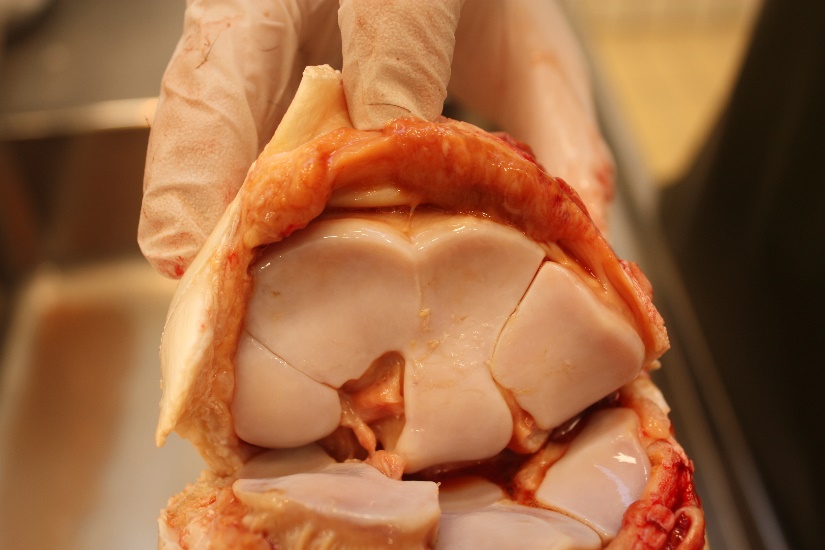


B.


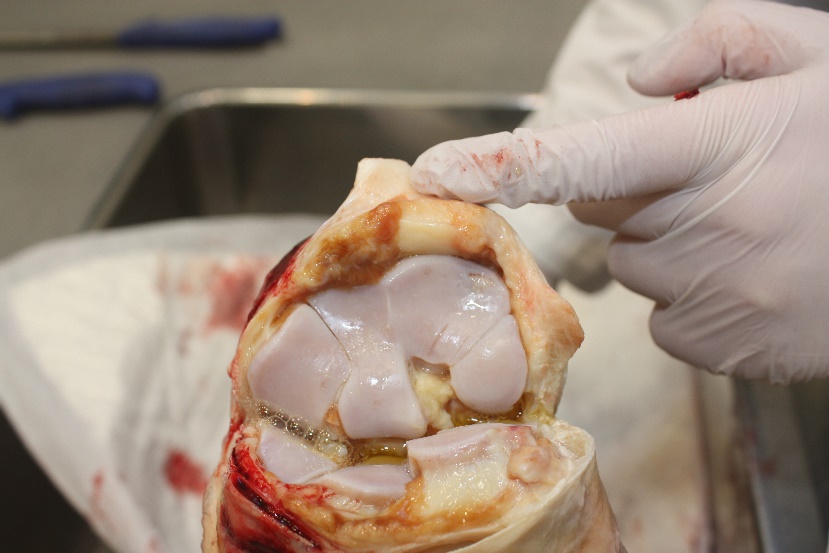


C.

D.
