# Supplementary material for: Small non-coding RNA landscape of extracellular vesicles from a post-traumatic model of equine osteoarthritis
Source: Front Vet Sci. 2022 Aug 8;9:901269. doi: 10.3389/fvets.2022.901269 (PMC9393553; doi:10.3389/fvets.2022.901269)

Supplementary File 2. Plasma-derived extracellular vesicle size and size distribution. (A) Mode, (B) D10, (C) D50 and (D) D90 size of extracellular vesicles isolated from plasma at intervals between 0-63 days following model induction. All analyses were conducted via nanoparticle tracking using a Nanosight NS300. Error bars ± 1 standard deviation.


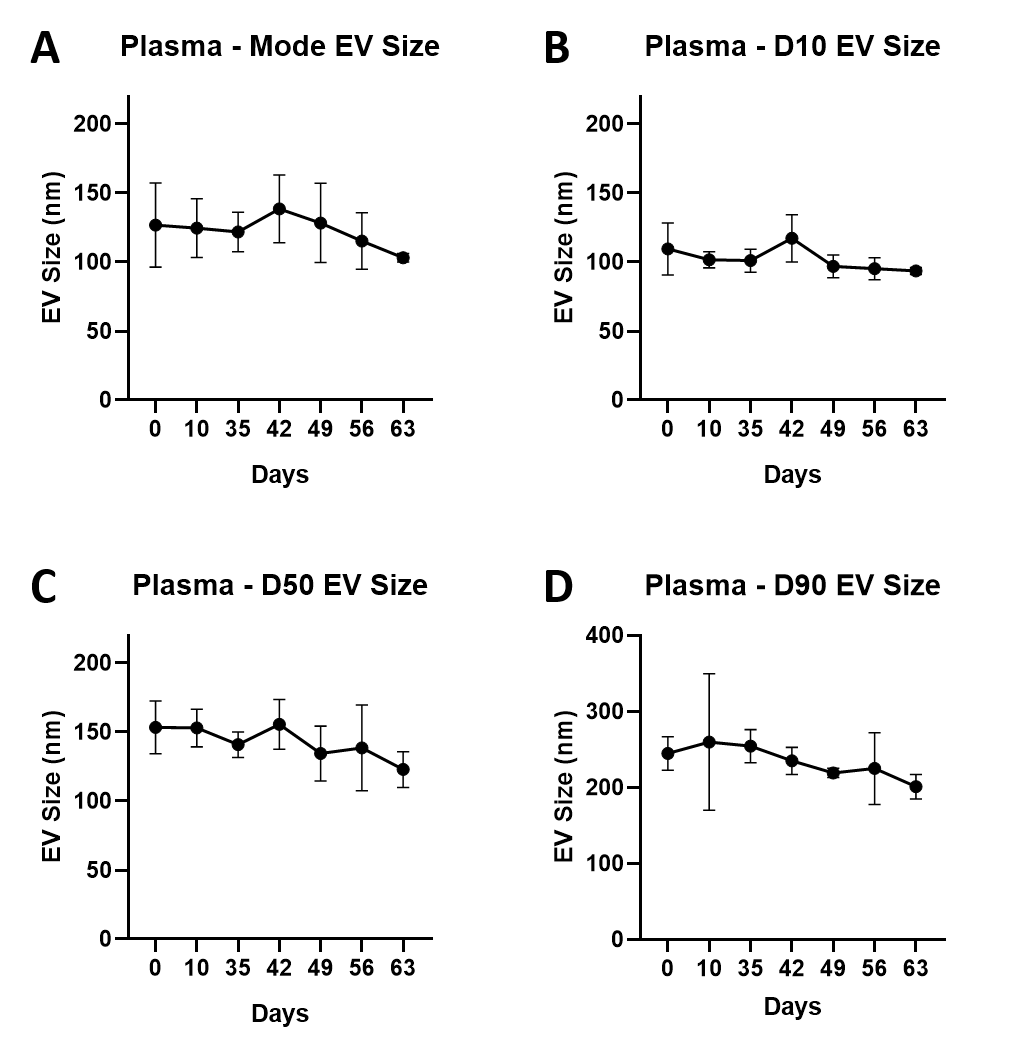

Supplement: Supplementary file 2 [file Table_2.DOCX]
