# Supplementary material for: Small non-coding RNA landscape of extracellular vesicles from a post-traumatic model of equine osteoarthritis
Source: Front Vet Sci. 2022 Aug 8;9:901269. doi: 10.3389/fvets.2022.901269 (PMC9393553; doi:10.3389/fvets.2022.901269)
Supplement: Supplementary file 4 [file Table_4.docx]

Supplementary File 4. Classes of non-coding RNAs identified in plasma (n= 4 horses, 6 time points, total 24 samples)-or synovial fluid (n= 4 horses, 6 time points, control and OA, total 48 samples) derived EVs following small RNA sequencing. RNA type was included if at least 30% of the samples in each group were identified.

| **Sample Type (group)** | **Type** | **Number** |
| --- | --- | --- |
| Plasma | lncRNA | 47 |
| Plasma | miRNA | 52 |
| Plasma | snoRNA | 6 |
| Plasma | snRNA | 17 |
| Plasma | tRNA | 36 |
| Synovial Fluid Control | lncRNA | 66 |
| Synovial Fluid Control | miRNA | 77 |
| Synovial Fluid Control | snoRNA | 4 |
| Synovial Fluid Control | snRNA | 18 |
| Synovial Fluid Control | tRNA | 51 |
| Synovial Fluid OA | lncRNA | 85 |
| Synovial Fluid OA | miRNA | 74 |
| Synovial Fluid OA | snoRNA | 4 |
| Synovial Fluid OA | snRNA | 20 |
| Synovial Fluid OA | tRNA | 50 |
