# Supplementary material for: Small non-coding RNA landscape of extracellular vesicles from a post-traumatic model of equine osteoarthritis
Source: Front Vet Sci. 2022 Aug 8;9:901269. doi: 10.3389/fvets.2022.901269 (PMC9393553; doi:10.3389/fvets.2022.901269)

Supplementary File 5. Differentially expressed miRNAs isolated from synovial fluid-derived extracellular vesicles. Error bars ± 1 standard deviation. ∗ = p < 0.05, ∗∗ = p < 0.01.


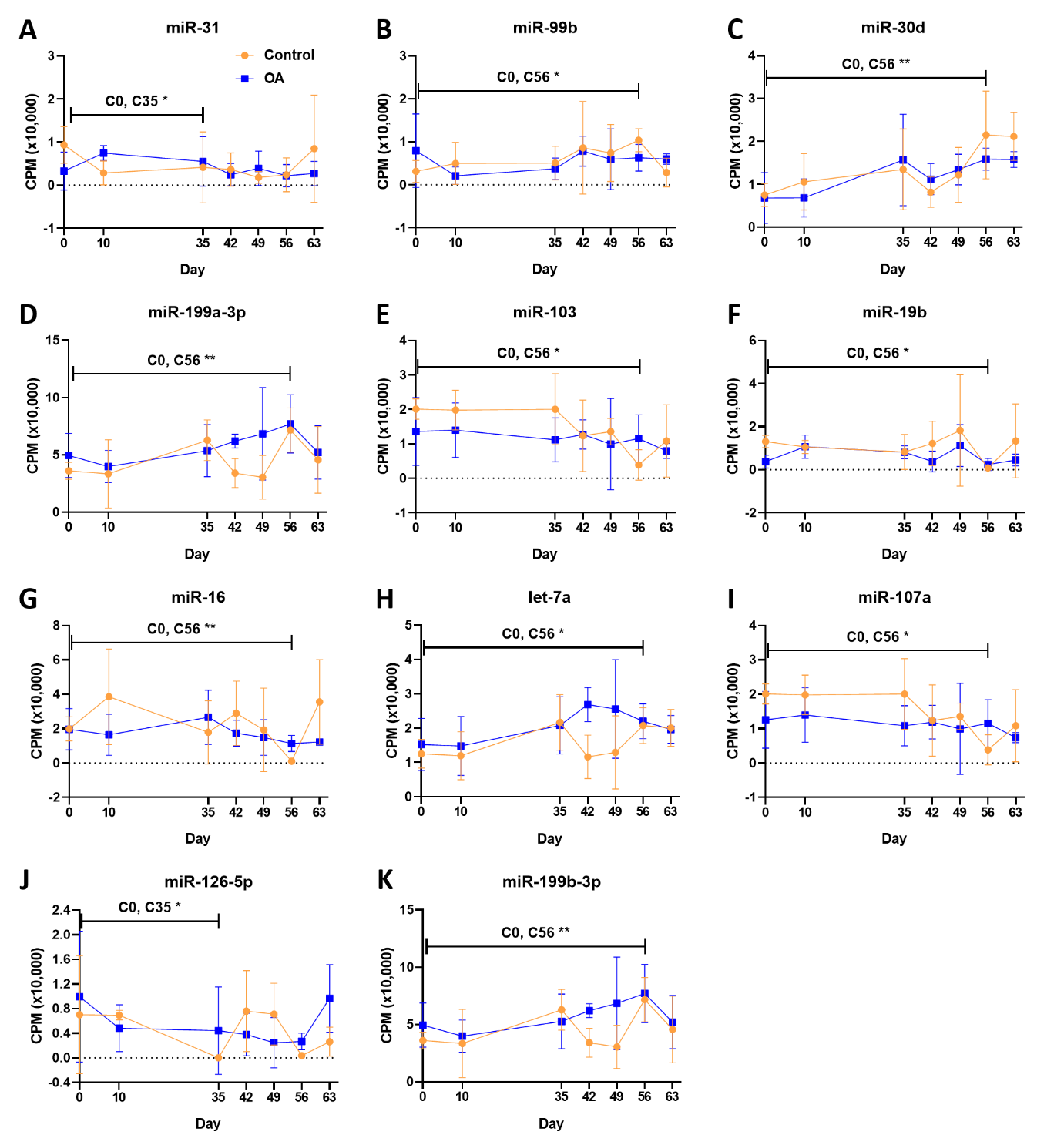

Supplement: Supplementary file 5 [file Table_5.DOCX]
